# Assessment of the Role of Niacin in Managing Cardiovascular Disease Outcomes: A Systematic Review and Meta-analysis

**DOI:** 10.1001/jamanetworkopen.2019.2224

**Published:** 2019-04-12

**Authors:** Elvira D’Andrea, Spencer P. Hey, Cherie L. Ramirez, Aaron S. Kesselheim

**Affiliations:** 1 Program on Regulation, Therapeutics, and Law (PORTAL) Biomarker Research Consortium, Division of Pharmacoepidemiology and Pharmacoeconomics, Department of Medicine, Brigham and Women’s Hospital, Harvard Medical School, Boston, Massachusetts

## Abstract

**Importance:**

Niacin remains a therapeutic option for patients with cardiovascular disease, but recent studies have called into question the effectiveness of other drugs that increase high-density lipoprotein cholesterol levels.

**Objective:**

To systematically review and evaluate the evidence supporting current US Food and Drug Administration–approved uses of niacin in cardiovascular disease prevention settings.

**Data Sources:**

MEDLINE, Embase, Cochrane Controlled Clinical Trial Register (Central), ClinicalTrials.gov, and TrialResults-center, from database inception to October 2017.

**Study Selection:**

The systematic review included clinical trials involving niacin as a treatment for cardiovascular disease. The meta-analysis included randomized clinical trials reporting niacin’s effect, as exposure, on at least 1 long-term cardiovascular disease outcome.

**Data Extraction and Synthesis:**

Aggregate study-level data were extracted between November 2017 and January 2018 by 3 independent reviewers, and the analysis was performed in February 2018. Inverse-variance weighted methods were used to produce pooled risk ratios using random-effects models for between-study heterogeneity. Random effects–weighted metaregression analysis was used to assess the association of change in high-density lipoprotein cholesterol levels with the log risk ratio of the pooled results.

**Main Outcomes and Measures:**

Cardiovascular disease, coronary heart disease mortality, and other cardiovascular events, including acute coronary syndrome, fatal and nonfatal stroke, revascularization, and major adverse cardiac events.

**Results:**

Of 119 clinical trials, 17 documented niacin’s effect on at least 1 cardiovascular disease outcome. The meta-analysis included 35 760 patients with histories of cardiovascular disease or dyslipidemia. Cumulative evidence found no preventive association of niacin with cardiovascular outcomes in secondary prevention. Stratified meta-analysis showed an association of niacin monotherapy with reduction of some cardiovascular events among patients without statin treatment (acute coronary syndrome: relative risk, 0.74; 95% CI, 0.58-0.96; stroke: relative risk, 0.74; 95% CI, 0.59-0.94; revascularization: relative risk, 0.51; 95% CI, 0.37-0.72). These results were mainly derived from 2 trials conducted in the 1970s and 1980s.

**Conclusions and Relevance:**

Niacin may have some use in lipid control for secondary prevention as monotherapy, perhaps in patients intolerant to statins, but evidence is from older studies on a population potentially not representative of current-day patients.

## Key Points

**Question:**

What is the evidence supporting the remaining US Food and Drug Administration–approved indications of niacin for patients with cardiovascular disease?

**Findings:**

In a systematic review and meta-analysis of 119 clinical trials that included 35 760 participants, 17 trials reported niacin’s effect on cardiovascular outcomes and did not suggest that niacin prevents cardiovascular disease overall. However, a stratified meta-analysis showed that niacin as monotherapy was associated with a reduction of some cardiovascular events, a result primarily derived from 2 trials conducted in the 1970s and 1980s.

**Meaning:**

Niacin may have a role as a monotherapy drug for lipid control in statin-intolerant patients, but, given substantial advancements in cardiovascular disease management since 1990, this indication should be restudied in current-day patients receiving usual baseline care.

## Teaser

This systematic review and meta-analysis evaluates the evidence supporting current US Food and Drug Administration–approved uses of niacin in cardiovascular disease prevention settings.

## Introduction

Niacin, also known as nicotinic acid or vitamin B_3_, has a convoluted history in the United States. It had been available for decades as an over-the-counter product, used as a vitamin supplement and to regulate lipid levels, although its use was limited by a common unpleasant adverse effect: flushing.^1^ In 1997, a prescription extended-release version of niacin offered a lower risk of flushing and was approved by the US Food and Drug Administration (FDA) for use in the secondary prevention of cardiovascular disease (CVD).^1^ The approved indications for the drug were later expanded to include managing primary hyperlipidemia and mixed dyslipidemia, reducing triglyceride levels, treating atherosclerotic disease in combination with bile acid–binding resin, and regulating lipid levels and preventing cardiovascular events in combination with 3-hydroxy-3-methylglutaryl–coenzyme A reductase inhibitors (statins).^2^ However, in 2016, the FDA withdrew the latter indication based on the results of 2 large prospective trials including 29 087 patients.^3,4^ These trials showed that adding niacin to statins did not improve prevention or reduce mortality. In addition, niacin is now no longer recommended by clinical guidelines to prevent CVD.^5^ Yet, prescription niacin retains its other FDA-approved indications and is still used by hundreds of thousands of US patients,^6^ while many others use the over-the-counter versions.

The concept that treatment with niacin may affect CVD (via a lipid modification pathway) was based on epidemiologic evidence dating back to the Framingham Heart Study, which identified an inverse relationship between high-density lipoprotein cholesterol (HDL-C) levels and the incidence of CVD.^7,8^ Plasma HDL-C levels were also predictive of recurrence and death in people who already experienced coronary events, suggesting a potential role of HDL-C in guiding secondary prevention treatment.^9,10^ This HDL-C hypothesis led to a growing interest in drugs that increase HDL-C levels, like niacin, as potential interventions to reduce the risk of CVD. Driven by the HDL-C biomarker hypothesis, use of the proprietary, prescription extended-release niacin increased by almost 200% from 2002 to 2009, such that by the end of 2009, niacin accounted for almost 700 000 prescriptions per month and $900 million in US annual expenditures,^11^ values that do not include those patients using the over-the-counter formulations. In 2013, when a generic version of extended-release niacin was introduced in the United States, worldwide sales exceeded $1 billion.^12^

However, evidence is now accumulating against the HDL-C hypothesis.^13,14,15,16^ Nevertheless, niacin retains several FDA-approved uses for patients with CVD.^2^ To evaluate the strength of evidence supporting these remaining uses of niacin, we performed a literature search and systematic review of all clinical trials testing niacin’s effects on lipid modification and cardiovascular risk as well as a meta-analysis to evaluate how the evidence evolved over time.

## Methods

Data collection took place between November 2017 and January 2018. We organized a systematic review to identify all clinical trials testing the effect of niacin therapy on cardiovascular risk. From this cohort of trials, we extracted those eligible for meta-analyses of niacin’s effects on cardiovascular outcomes and metaregression analyses on the association of change in HDL-C level with CVD morbidity and mortality. Data analysis was performed in February 2018. Because it did not involve primary data collection, the protocol was not submitted for institutional review board approval and did not require informed consent. Data reporting following the Preferred Reporting Items for Systematic Reviews and Meta-analyses (PRISMA) reporting guideline.^17^

### Systematic Review

We searched MEDLINE, Embase, Cochrane Controlled Clinical Trial Register (Central), ClinicalTrials.gov, and TrialResults-center for clinical trials involving niacin as a treatment for CVD from database inception to October 2017. The search strings were based on the drug (niacin OR nicotinic acid OR Niaspan OR nicotinic acid derivatives), effectiveness outcomes (mortality OR cardiovascular disease* OR coronary heart disease OR myocardial infarction OR coronary artery disease OR coronary disease OR acute coronary syndrome OR stroke OR heart failure OR revascularization OR congestive heart disease OR cholesterol OR HDL OR triglycerides OR LDL OR hypercholesterolemia OR atherosclerosis OR dyslipidemia), and study design (clinical trial). The search was restricted to English-language, Italian-language, and Spanish-language articles, based on coauthor language abilities. Reference lists of included studies were screened.

Three of us (E.D., S.P.H., and C.L.R.) independently removed duplicates and reviewed titles and abstracts for potentially relevant articles. We sought clinical trials comparing nicotinic acid as monotherapy or combined with other agents with placebo, conventional therapy, or other lipid-lowering interventions (eg, statins, diet). We excluded studies if they targeted populations with competing CVD risks that would limit the generalizability of the findings (eg, chronic kidney disease,^18^ diabetes,^19^ HIV^20^). Discrepancies were resolved by consensus among the reviewers or, if needed, all authors. Reasons for study exclusion were recorded (eTable 1 in the Supplement).

We extracted information about authors, year of publication, duration of drug exposure, sample size, and outcome. We separately categorized trials in which the investigators evaluated only surrogate measures (such as increase in HDL-C level) vs those that included at least 1 clinical CVD outcome (eTable 2 in the Supplement).

### Meta-analysis

To conduct a meta-analysis, we included trials from within our main cohort that were randomized, had a control group in which the arms differed with respect to the presence of niacin therapy (eg, niacin vs placebo, statin-niacin vs statin), had a follow-up period of at least 6 months (minimum time frame in which effects on CVD are expected to emerge),^21,22^ and reported at least 1 outcome related to cardiovascular mortality (ie, CVD mortality and coronary heart disease mortality) or other cardiovascular outcomes (ie, acute coronary syndrome, cerebrovascular events, revascularization procedures, and a composite of major adverse cardiovascular events) separately for each study group (eTable 2 in the Supplement). Among this subset of trials, we extracted basic features (ie, country, blinding, niacin formulation, intervention regimen and dosage, and control regimen and dosage), participant information (ie, study population, age, and sex), and outcomes (ie, cases vs no cases for each CVD outcome and lipid measurements at the baseline and at the end of follow-up in experimental and control groups). If trials failed to report exact lipid concentrations in the text, we extracted those data from graphs or attempted to contact the corresponding author. If a trial compared different doses of niacin, we extracted the data on the dosage recommended by the FDA or in clinical guidelines. Outcomes data were identified at the time of last reported patient follow-up.

For this set of studies, 2 of us (E.D. and C.L.R.) independently evaluated the methodological quality of each trial from the Cochrane Handbook for Systematic Reviews of Interventions based on randomization (generation of allocation sequences and concealment of allocation), blinding, adequacy of analyses (including dropouts and withdrawals),^23^ and selective reporting of outcomes.^24^ Disagreement was resolved as before. The results on methodologic quality are presented in eTable 3 in the Supplement.^23^

Using Review Manager version 5.3 (the Cochrane Collaboration) and Stata version 15 (StataCorp), we analyzed the data with a random-effects model, calculating the relative risk (RR).^25^ Trials with no events in both arms, which differed with respect to the presence of niacin therapy, were excluded.^26,27^ We assessed heterogeneity and evaluated potential sources of heterogeneity by eliminating 1 trial in turn.^28^ In further sensitivity analyses, we explored the influence of risk of bias on the outcomes, excluding trials with high or unclear bias. Finally, we detected the influence of individual studies on the summary of the effect estimate of each CVD outcome. A subgroup analysis was performed to explore the effect of niacin with and without statins.

### Metaregression Analysis

Within the studies eligible for meta-analysis, we excluded those that did not report HDL-C measurements at baseline and at the end of drug exposure. We then used random effects–weighted metaregression analysis to assess the association of difference in change in HDL-C levels for niacin and control groups with the log risk ratio of the CVD outcomes of the meta-analysis.^29^ We ran univariate analyses and multivariate analyses including the covariates that can influence the effect of HDL-C levels on cardiovascular outcomes (change in low-density lipoprotein cholesterol [LDL-C] levels and sample size).^30,31^ While the analyses are presented with percentage change in HDL-C level subfraction, results were consistent with those from absolute change in milligrams per deciliter or millimoles per liter.

## Results

**Figure 1.**
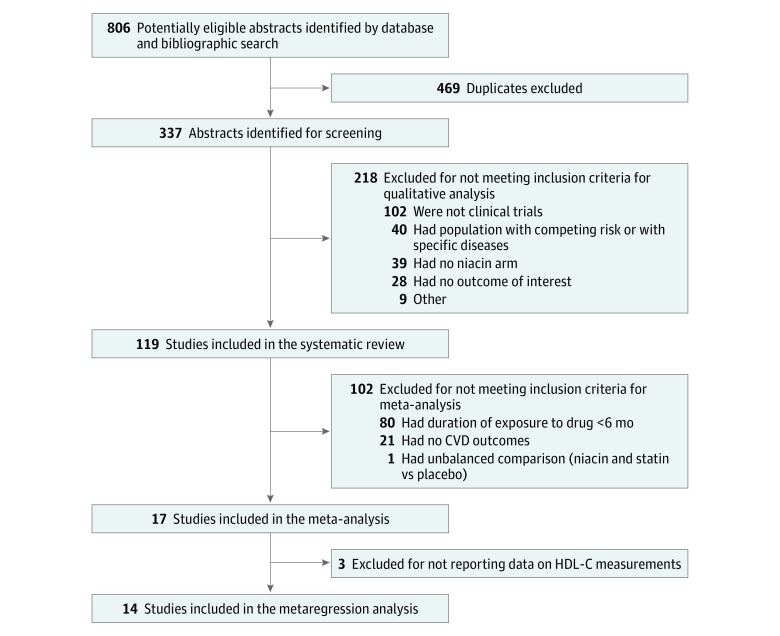
Flow Diagram of Included Studies for the Systematic Review, Meta-analysis, and Metaregression Analysis CVD indicates cardiovascular disease; HDL-C, high-density lipoprotein cholesterol.

**Figure 2.**
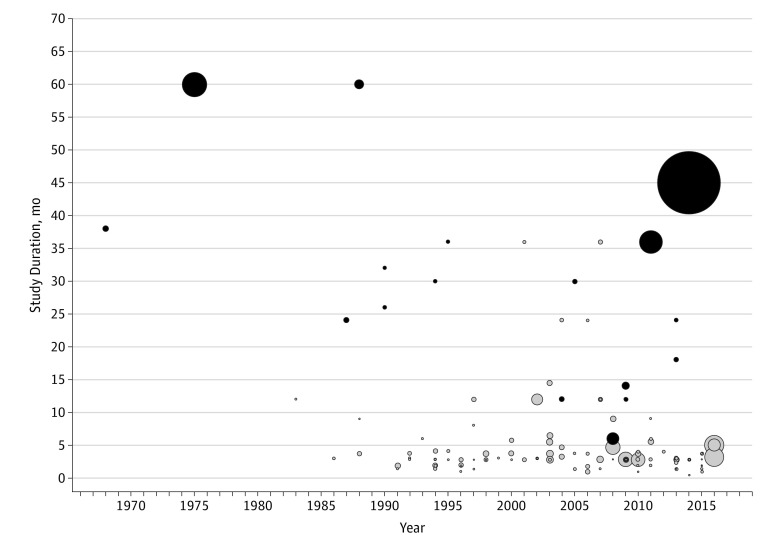
Scatterplot of Randomized Clinical Trials Included in Systematic Review The plot shows publication over time relative to duration of the randomized clinical trials included in our systematic review. Black circles represent included trials, which provided information on long-term cardiovascular outcomes, while the gray circles represent the others, which provided information only on surrogate measures and/or had a follow-up period shorter than 6 months. The size of the circles varies according to the sample size of each trial.

We identified 119 clinical trials for our systematic review (Figure 1). Seventeen trials (14.3%) documented niacin’s effect on a CVD outcome,^3,4,31,32,33,34,35,36,37,38,39,40,41,42,43,44,45^ while the remaining 102 (85.7%) based their conclusions on surrogate measures only, mainly increases in HDL-C levels or decreases in LDL-C or triglyceride levels (Figure 2). Among the 17 studies with CVD information, only 6 trials included 1 or more cardiovascular event as a study outcome.^3,4,31,32,40^ Overall, 87 studies (73.1%) targeted populations with dyslipidemia, with or without a history of cardiovascular events; 26 (21.8%) with coronary events and/or atherosclerotic progression; and 6 (5.0%) hybrid populations or patients with other diseases.

### Meta-analysis

The 17 studies that provided data on CVD outcomes included 35 760 patients, with 17 105 individuals (47.8%) randomly assigned to niacin arms and 18 655 individuals (52.2%) receiving placebo, usual therapy, or other lipid-lowering agents (Table 1). Six studies presented an overall low risk of bias,^3,4,32,35,39,40^ while 4 were at overall high risk of bias^34,37,38,43^ (eTable 3 in the Supplement). The most common bias was attrition with unavailable outcome data.

**Table 1.** General Characteristics of the 17 Included Randomized Clinical Trials

Source; Country	RCT Name	Study Design, Center, Blinding	Follow-up, mo	Regimen and Dosage, g/d		Total Sample (Intervention, Control), No.	Population Target	Age, Mean (SD), y		Male, No. (%)
				Intervention	Control			Intervention	Control	
Schoch,^31^ 1969; United States	VA Drug	Factorial, single center, double blind	38	IR-niacin, 4	Placebo	220 (77, 143)	History of MI	53.7 (NR)	53.7 (NR)	220 (100)
CDP,^32^ 1975; United States	CDP	Parallel, multicenter, double blind	60	IR-niacin, 3	Lactose placebo	3908 (1119, 2789)	History of MI	45 (NR)	43 (NR)	3908 (100)
Blankenhorn et al,^33^ 1987; United States	CLAS I	Parallel, single center, double blind	24	IR-niacin, 4.3 + colestipol, 30^a^	Placebo	188 (94, 94)	History of CABG, atherosclerosis	53.9 (4.85)	54.5 (4.85)	188 (100)
Carlson and Rosenhamer,^34^ 1988; Sweden	STOCKHOLM	Parallel, single center, open label	60	IR-niacin, 3 + clofibrate, 2	Placebo	555 (279, 276)	History of MI	60.7 (NR)	61.1 (NR)	442 (79.6)
Brown et al,^35^ 1990; United States	FATS	Parallel, multicenter, double blind	32	IR-niacin, 4 + colestipol, 30	Placebo + colestipol, 30	100 (48, 52)	History of coronary atherosclerosis	47 (NR)	47 (NR)	100 (100)
Kane et al,^36^ 1990; United States	UCSF-SCOR	Parallel, multicenter, open label	26	IR-niacin, 7.5 + colestipol, 15-20	Placebo + colestipol, 15-20	97 (48, 49)	hFH and history of atherosclerosis	41.4 (12)	42.4 (13)	42 (43)
Sacks et al,^37^ 1994; United States	HARP	Parallel, single center, single blind	30	SR-niacin, 1.5-3 + gemfibrozil, 0.6-1.2 and cholestyramine, 8-16	Placebo + diet	79 (40, 39)^b^	History of CHD and atherosclerosis	57 (8)	59 (9)	70 (89)^b^
Caruzzo et al,^38^ 1995; United States	PAST	Parallel, single center, open label	36	Acipimox, 0.5 + diet	Diet	85 (40, 45)	Hyperlipemia, atherosclerosis, and/or previous MI	51 (2.8)	51 (2.8)	81 (95)
Taylor et al,^39^ 2004; United States	ARBITER-2	Factorial, single center, double blind	12	ER-niacin, 1 + any statin, NR	Placebo + any statin, NR	167 (87, 80)	History of CHD and statin therapy	67 (10)	68 (10)	152 (91.0)
Whitney et al,^40^ 2005; United States	AFREGS	Parallel, single center, double blind	30	IR-niacin, 2.5 + gemfibrozil, 1.2 ± cholestyramine, 8.4^a^	Placebo ± cholestyramine, NR	143 (71, 72)	Low HDL-C levels and history of CHD	63.3 (7.5)	63.1 (6.8)	132 (92.3)
Guyton et al,^41^ 2008; United States	NA	Parallel, multicenter, double blind	6	ER-niacin, 2 + simvastatin, 0.01 and ezetimibe, 0.02	Placebo + simvastatin, 0.01 and ezetimibe, 0.02	603 (391, 212)^b^	Type IIa or IIb hyperlipidemia	56.9 (10.9)	57.5 (10.3)	472 (49.8)
Taylor et al,^42^ 2009; United States	ARBITER 6-HALTS	Parallel, single center, open label	14	ER-niacin, 2 + any statin, NR	Ezetimibe 0.01 and any statin, NR	208 (97, 111)^b^	History of CHD and statin therapy	64 (11)	65 (11)	167 (80.3)^b^
Sang et al,^43^ 2009; United States	NA	Parallel, single center, NR	12	ER-niacin, 1 + atorvastatin, 0.01	Atorvastatin, 0.01	108 (52, 56)	High total cholesterol and atherosclerosis	72.9 (6.9)	68.8 (10.0)	66 (61)
Boden et al,^3^ 2011; United States and Canada	AIM-HIGH	Parallel, multicenter, double blind	36	ER-niacin, 1.5-2 + simvastatin, 0.04-0.08 ± ezetimibe	Placebo + simvastatin, 0.04-0.08 ± ezetimibe	3414 (1718, 1696)	History of CHD and dyslipidemia^c^	63.7 (8.8)	63.7 (8.8)	2910 (85.2)
Sibley et al,^44^ 2013; United States	NIA Plaque	Parallel, single center, double blind	18	ER-niacin, 1.5 + any statin, 0.021	Placebo + any statin, 0.021	117 (59, 58)^b^	History of CHD and atherosclerosis	73 (NR)	72 (NR)	118 (81.4)
Brunner et al,^45^ 2013; United States	ELIMIT	Parallel, multicenter, double blind	24	ER-niacin, 1.5 + simvastatin, 0.04 and ezetimibe, 0.01	Placebo + simvastatin, 0.04	95 (47, 48)^b^	Dyslipidemia, hypertension, or diabetes and history of PAD^c^	62.1 (7.8)	63.9 (7.1)	89 (94)^b^
Landray et al,^4^ 2014; United Kingdom, Scandinavia, and China	HPS2-THRIVE	Parallel, multicenter, double blind	45	ER-niacin, 2 + simvastatin, 0.04 and laropiprant, 0.04	Placebo + simvastatin, 0.04	25 673 (12 838, 12 835)	History of CHD, PAD, or diabetes	64.9 (7.5)	64.9 (7.5)	21 229 (82.7)

Abbreviations: AFREGS, Armed Forces Regression Study; AIM-HIGH, Atherothrombosis Intervention in Metabolic Syndrome With Low HDL/High Triglycerides trial; ARBITER-2, Arterial Biology for the Investigation of the Treatment Effects of Reducing Cholesterol trial; ARBITER 6-HALTS, Arterial Biology for the Investigation of the Treatment Effects of Reducing Cholesterol 6-HDL and LDL Treatment Strategies in Atherosclerosis trial; CDP, Coronary Drug Project; CABG, coronary artery bypass grafting; CHD, coronary heart disease; CLAS I, Cholesterol-Lowering Atherosclerosis Study I; ELIMIT, Effect of Lipid Modification on Peripheral Artery Disease After Endovascular Intervention Trial; ER, extended release; FATS, Familial Atherosclerosis Treatment Study; HARP, Heart and Renal Protection study; HDL-C, high-density lipoprotein cholesterol; hFH, heterozygous familial hypercholesterolemia; HPS2-THRIVE, Heart Protection Study 2–Treatment of HDL to Reduce the Incidence of Vascular Events; IR, immediate release; MI, myocardial infarction; NA, not applicable; NIA Plaque, National Institute on Aging Plaque Study; NR, not reported; PAD, peripheral artery disease; PAST, Prevenzione Aterosclerosi Studio Torino; RCT, randomized clinical trial; SR, sustained release; STOCKHOLM, Stockholm Ischaemic Heart Disease Secondary Prevention Study; UCSF-SCOR, University of California San Francisco–Arteriosclerosis Specialized Center of Research Intervention Trial; VA Drug, Veterans Affairs drug study.

^a^ Maximum dosage achieved in the titration process.

^b^ Numbers include only participants who completed the treatment originally allocated.

^c^ Low HDL-C levels (<50 mg/dL; to convert to millimoles per liter, multiply by 0.0259), elevated triglyceride levels (150-400 mg/dL; to convert to millimoles per liter, multiply by 0.0113), and small and dense particles of LDL-C (<180 mg/dL; to convert to millimoles per liter, multiply by 0.0259).

**Figure 3.**
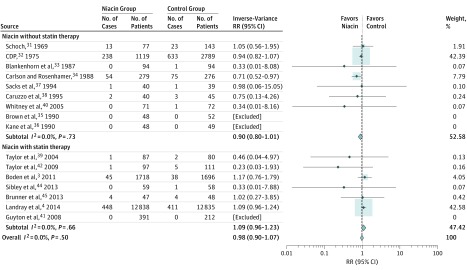
Forest Plots of Meta-analyses on the Effect of Niacin Therapy on Cardiovascular Disease Mortality The number of events by allocated treatment and the point estimates of the effect sizes are shown for individual trials and subgroups of trials based on the presence of statin as background therapy. Weights are from random-effects analysis. Risk ratios (RRs) for individual trials or subgroups of trials are indicated by squares and 95% CIs by horizontal lines. Pooled estimates and their 95% CIs are represented by diamonds. The size of the squares and the diamonds are proportional to the weight assigned to the relative effect sizes. CDP indicates Coronary Drug Program.

The meta-analysis showed no association of niacin with CVD mortality (RR, 0.98; 95% CI, 0.90-1.07) or coronary heart disease mortality (RR, 0.90; 95% CI, 0.76-1.06) in patients with a history of coronary disease, atherosclerosis, or dyslipidemia (Figure 3; eFigure 1 in the Supplement). There was also no significant association of niacin treatment with stroke (RR, 0.95; 95% CI, 0.85-1.06), acute coronary syndrome (RR, 0.87; 95% CI, 0.74-1.02), or the combined end point of major adverse cardiac events (RR, 0.88; 95% CI, 0.76-1.01). These results were consistent with those obtained in the subgroup with statin cotreatment.

In the cumulative meta-analysis, the estimates initially described a preventive association of niacin with cardiovascular outcomes, owing to results from the Coronary Drug Project study (1975)^32^ and the Stockholm Ischaemic Heart Disease Secondary Prevention Study (1988)^34^ (eFigure 2 in the Supplement). The 9 studies published from 1990 to 2011 contributed little further predictive value to the cumulative estimates.^35,36,37,38,39,40,41,42,43,44^ The findings then moved toward the null since 2011, owing to the Atherothrombosis Intervention in Metabolic Syndrome With Low HDL/High Triglycerides trial (AIM-HIGH, 2011)^3^ and Heart Protection Study 2–Treatment of HDL to Reduce the Incidence of Vascular Events (HSP2-THRIVE, 2014)^4^ trials (eFigure 2 in the Supplement).

### Meta-analysis on Subgroup of Patients Without Statin Treatment

In the subgroup of patients not treated with a statin, niacin arms showed improvement on secondary outcomes measurements. For example, among trials reporting data on acute coronary syndrome,^3,4,31,32,33,34,35,36,37,38,39,40,41,42,43,44,45^ the niacin arms were associated with a 26% lower rate (RR, 0.74; 95% CI, 0.58-0.96) compared with controls among patients not treated with statins. As another example, niacin treatment was associated with a 26% reduction in stroke events (RR, 0.74; 95% CI, 0.59-0.94) in the subgroup without costatin treatment. Finally, in the 13 trials that measured risk of revascularization procedure,^3,4,32,33,35,37,38,39,40,42,43,44,45^ niacin treatment was associated with reduced risk (RR, 0.79; 95% CI, 0.64-0.98) for both groups. The reduction point estimate was lower in the subgroup of patients without a background statin treatment (RR, 0.51; 95% CI, 0.37-0.72) compared with the subgroup of patients with statin treatment (RR, 0.91; 95% CI, 0.84-0.99). Among other clinical outcomes, such as CVD, coronary heart mortality, and major adverse cardiac events, the associations were directionally similar but not significant (Figure 3 and eFigure 1 in the Supplement).

### Metaregression Analysis

Overall, 14 studies provided data with which to analyze the association of the change in HDL-C levels with cardiovascular outcomes.^3,4,33,35,36,37,38,39,40,41,42,43,44,45^ Univariate and multivariate metaregression analyses, adjusted for change in LDL-C measurements and sample size, are presented in Table 2. Change in HDL-C levels was not associated with the log risk ratio for primary cardiovascular outcomes. Change in HDL-C levels does not appear to explain differences in CVD and coronary heart disease mortality, acute coronary syndrome, stroke, revascularization procedures, or major adverse cardiac events.

**Table 2.** Metaregression Models Investigating Association of Change in HDL-C Levels With Log Risk Ratios of Clinical Outcomes

Regression Model	Change in Risk per 1% Increase in HDL-C (95% CI)	SE	*P* Value
CVD mortality			
Univariable	−0.028 (−0.105 to 0.049)	0.035	.44
Multivariable^a^	−0.028 (−0.113 to 0.058)	0.038	.48
Coronary heart disease mortality			
Univariable	−0.047 (−0.205 to 0.111)	0.061	.47
Multivariable^a^	−0.025 (−0.268 to 0.218)	0.076	.76
Acute coronary syndrome			
Univariable	−0.017 (−0.084 to 0.050)	0.031	.60
Multivariable^a^	−0.034 (−0.125 to 0.057)	0.040	.41
Cerebrovascular events			
Univariable	−0.068 (−0.201 to 0.065)	0.058	.27
Multivariable^a^	−0.075 (−0.242 to 0.091)	0.068	.31
Revascularization procedures			
Univariable	−0.016 (−0.061 to 0.029)	0.021	.44
Multivariable^a^	−0.020 (−0.090 to 0.049)	0.030	.52
Any CVD events			
Univariable	−0.008 (−0.042 to 0.025)	0.015	.59
Multivariable^a^	−0.014 (−0.062 to 0.035)	0.022	.54

Abbreviations: CVD, cardiovascular disease; HDL-C, high-density lipoprotein cholesterol.

^a^ Models include adjustment for low-density lipoprotein cholesterol level and sample size.

## Discussion

Our systematic review found that most clinical trials conducted over the last 60 years to assess the effectiveness of niacin in cardiovascular prevention settings evaluated changes in surrogate measures, mainly HDL-C, LDL-C, and triglyceride levels. Among trials qualifying for the meta-analysis, niacin was not associated with a reduced risk of cardiovascular morbidity and mortality for all patients, with similar results among the subgroup of statin-treated patients. These findings are consistent with quantitative syntheses from previous meta-analyses.^46,47^ By contrast, pooled estimates of the trials performed on the subgroup of patients without concurrent statin therapy indicated an association of niacin with 3 clinical outcomes, often reported as secondary end points: a reduced risk for acute coronary syndrome, stroke, and revascularization procedures.

Prescription extended-release niacin has FDA-approved indications as monotherapy for treating primary hyperlipidemia and mixed dyslipidemia. While many trials have been conducted in patients with dyslipidemia, showing favorable changes in lipid profiles, few reported information on cardiovascular outcomes,^3,36,38,41,43,45^ and even fewer were designed to detect changes in these clinical outcomes.^3^ Therefore, evidence that using niacin alone to correct primary hyperlipidemia or mixed dyslipidemia to change the risk of cardiovascular mortality or morbidity is limited.

Niaspan also retains an FDA-approved indication as monotherapy in secondary prevention to reduce recurrences of nonfatal myocardial infarction.^2^ In stratified analyses, we found that niacin is associated with some positive outcomes in this clinical situation among patients who are not treated with statins. Despite the widespread availability of low-cost, high-potency statins in the current market, some patients may still experience intolerable adverse effects or want to avoid potential drug-drug interactions. Based on our findings, niacin may be a reasonable clinical choice in these cases, but the results from these subgroup analyses were mainly derived from the Coronary Drug Project (1975),^32^ the study that also serves as the main reference in the FDA labeling for this specific indication (Cholesterol-Lowering Atherosclerosis Study I [1987]^36^ and Familial Atherosclerosis Treatment Study [1990]^35^ are also cited in the labeling), and the Stockholm Ischaemic Heart Disease Secondary Prevention Study (1988).^34^ Thus, results supporting this indication are based on a target population that may not be generalizable to the current population receiving usual care. Apart from the introduction of statin therapy, other changes in the last 30 years to prevent cardiovascular episodes include more widespread use of aspirin, antiplatelet therapy, and β-blockers for patients with previous myocardial infarction as well as inhibitors of the renin-angiotensin system. The aggregate effects of these interventions might have changed the underlying risk for cardiovascular events even among patients not taking statins and, consequently, reduced the marginal benefit that niacin might provide for contemporary patients. We therefore recommend that the FDA convene an advisory committee to reconsider this approved indication for niacin products, leading to a new trial, perhaps funded by the National Heart, Lung, and Blood Institute, to confirm that it remains relevant.

An additional concern is the over-the-counter use of niacin for cardioprotection. In this context, any incremental benefits of niacin as monotherapy become even more indeterminate, especially because the dosage of the over-the-counter formulation is substantially lower than the cardioprotective regimen administered in the clinical trials. This inappropriate use might also be associated with an increase in the risk of adverse events without an improvement in outcomes.

Other important limitations to the clinical use of niacin are the adverse events. Cutaneous flushing is a well-known adverse effect, and it has been recognized as the major reason for the discontinuation of niacin therapy, with rates as high as 25% to 40%.^48,49^ More serious adverse effects, such as gastrointestinal events, liver toxic effects, and musculoskeletal damage, have also been associated with the use of niacin.^50^

Our findings add further evidence against the clinical hypothesis that increasing HDL-C levels may play a key part in modifying cardiovascular risk. Biomarkers, like HDL-C levels, and other surrogate measures that are validated to accurately predict clinical outcomes, such as high blood pressure and cardiovascular mortality, can improve the efficiency and expediency of drug development because changes to surrogate measures often can be observed sooner or more easily.^51^ As a result, such changes are now frequently used by the FDA as the basis for new drug approvals.^52^ However, some surrogate measures have been found in later testing to not have the expected clinical benefits or to have higher rates of adverse events.^53^ If surrogate measures are not known to correlate with clinical outcomes, we should be wary about using them to guide prescribing decisions or as end points in clinical trials. In line with previous analyses on niacin^19^ and on other HDL-C level–increasing agents, such as cholesteryl ester transfer protein inhibitors,^54^ evidence is accumulating that the HDL-C level is not a sensitive indicator of cardiovascular risk modification, clouding its use as a surrogate measure in clinical research or clinical practice. With our stratified analysis, we were able to show that when the LDL-C level is corrected using statins, there is no evidence that adding niacin provides incremental clinical benefit, which in such a clinical scenario should be mainly because of its ability to increase HDL-C levels. The metaregression also showed no association of change in HDL-C levels with cardiovascular outcomes.

The evolution of knowledge about niacin can also help cardiovascular trial investigators and policymakers search for the next generation of cardiovascular treatments. We found that the Coronary Drug Project (1975)^32^ played the central part in initially defining the association of niacin with cardiovascular risk reduction. The Stockholm Ischaemic Heart Disease Secondary Prevention Study (1988)^34^ also influenced the trend of the cumulative estimates (although it was a poor-quality trial), accentuating niacin’s protective action for certain cardiovascular outcomes. After 1988, numerous trials were conducted and published but contributed little more than the existing trials to change the evolving connection between niacin treatment and CVD. It was not until the 2011 AIM-HIGH^3^ and 2014 HSP2-THRIVE^4^ trials that the role of niacin in cardiovascular prevention, at least as add-on therapy, was clarified. In the 4-decade gap between the publication of the Coronary Drug Project^32^ and the AIM-HIGH^3^ and HSP2-THRIVE^4^ trials, the clinical efficacy of niacin remained uncertain despite the investment of substantial human and financial resources in these trials. Such trials can divert limited research resources from potentially more useful purposes.^55,56^ Many of these trials in retrospect were unhelpful because they repeatedly retested niacin’s effect on surrogate measures, including lipid biomarkers such as HDL-C level, without formal validation that these biomarkers were clinically useful. Better oversight about the validity and use of biomarkers in clinical trials may have helped guide resources to trials of HDL-C levels and niacin that would have contributed to evolving knowledge.^57,58^

To our knowledge, this is the first systematic review that quantifies the number of trials performed on niacin’s association with CVD prevention, finding that most of these rely on surrogate measures, and only 3 trials provided high-quality evidence. Compared with other meta-analyses,^2,13,14,15,16,47^ this was also the first that investigated the strength of evidence behind the FDA-approved indications of niacin as monotherapy in cardiovascular prevention, finding these to be insufficient and not generalizable.

### Limitations

Our meta-analysis has several limitations, mostly owing to differences between the included studies. Sex, age, mean lipid values at study entry, treatment dosage, and duration of follow-up differ among studies. In a few clinical trials, the niacin arm was a combination of niacin and fibrate or bile acid sequestrants.^35,36,41^ However, a sensitivity analysis removing those trials showed unchanged results. Another limitation is the risk of ecological bias in the metaregression analysis. Because we assessed the association between 2 individual-level variables rather than trial-level variables, the results are less robust and at higher risk of bias.

## Conclusions

Widespread perceptions about the use of prescription drugs may change when treatments originally guided by surrogate measures—as in the case of niacin and HDL-C levels—are then studied in adequately powered trials of clinical outcomes. The role of niacin as add-on therapy to statin treatment based on its effect on HDL-C levels was clarified in 2011 and 2014, when 2 large trials showed a lack of clinical effectiveness. Yet niacin retains an FDA-approved indication as monotherapy for treating dyslipidemia, a main risk factor for cardiovascular events and myocardial infarction. We found some evidence of clinical benefit in this context, although this was based on older trials with study populations likely to differ from contemporary patients in terms of underlying cardiovascular risk. Further prospective trials of niacin are needed to resolve this question and determine what role it may have in the current range of therapies intended to manage CVD.

## Notes

Supplement. **eTable 1.** List of all excluded studies after removing duplicates and reasons for exclusions **eTable 2.** Definitions of cardiovascular outcomes evaluated in the meta-analyses and meta-regression analysis **eTable 3.** Quality assessment and risk of bias of the studies included in the meta-analyses and meta-regression analysis **eFigure 1.** Forest plot of meta-analyses on the effect of niacin therapy over time on coronary heart disease mortality, acute coronary syndrome, revascularization procedures, and MACE **eFigure 2.** Forest plot of cumulative meta-analyses on the effect of niacin therapy over time on the CVD mortality, coronary heart disease mortality, acute coronary syndrome, revascularization procedures, and MACE
