# Supplementary material for: Assessment of the Role of Niacin in Managing Cardiovascular Disease Outcomes: A Systematic Review and Meta-analysis
Source: JAMA Netw Open. 2019 Apr 12;2(4):e192224. doi: 10.1001/jamanetworkopen.2019.2224 (PMC6481429; doi:10.1001/jamanetworkopen.2019.2224)
Supplement: Supplement. — eTable 1. List of all excluded studies after removing duplicates and reasons for exclusions eTable 2. Definitions of cardiovascular outcomes evaluated in the meta-analyses and meta-regression analysis eTable 3. Quality assessment and risk of bias of the studies included in the meta-analyses and meta-regression analysis eFigure 1. Forest plot of meta-analyses on the effect of niacin therapy over time on coronary heart disease mortality, acute coronary syndrome, revascularization procedures, and MACE eFigure 2. Forest plot of cumulative meta-analyses on the effect of niacin therapy over time on the CVD mortality, coronary heart disease mortality, acute coronary syndrome, revascularization procedures, and MACE [file jamanetwopen-2-e192224-s001.pdf]

Supplementary Online Content

D'Andrea E, Hey SP, Ramirez CL, Kesselheim AS. Assessment of the role of niacin in managing cardiovascular disease outcomes: a systematic review and meta-analysis. *JAMA Netw Open.* 2019;2(4):e192224. doi:10.1001/jamanetworkopen.2019.2224

eTable 1. List of all excluded studies after removing duplicates and reasons for exclusions
eTable 2. Definitions of cardiovascular outcomes evaluated in the meta-analyses and meta-regression analysis
eTable 3. Quality assessment and risk of bias of the studies included in the meta-analyses and meta-regression analysis
eFigure 1. Forest plot of meta-analyses on the effect of niacin therapy over time on coronary heart disease mortality, acute coronary syndrome, revascularization procedures, and MACE
eFigure 2. Forest plot of cumulative meta-analyses on the effect of niacin therapy over time on the CVD mortality, coronary heart disease mortality, acute coronary syndrome, revascularization procedures, and MACE

This supplementary material has been provided by the authors to give readers additional information about their work.

© 2019 D'Andrea E et al. *JAMA Network Open.*

eTable 1. List of all excluded studies after removing duplicates and reasons for exclusions

n.	References	Reason(s) for exclusion from qualitative and quantitative analysis
1	Ballantyne CM, Shah S, Kher U, Hunter JA, Gill GG, Cressman MD, Ashraf TB, Johnson-Levonas AO, Mitchel YB. Lipid-Modifying Efficacy and Tolerability of Anacetrapib Added to Ongoing Statin Therapy in Patients with Hypercholesterolemia or Low High-Density Lipoprotein Cholesterol. Am J Cardiol. 2017 Feb 1;119(3):388-396. PubMed PMID: 27956003. registered on ClinicalTrials.gov NCT01717300	No niacin
2	Silva de Paula E, Carneiro MF, Grotto D, Hernandes LC, Antunes LM, Barbosa F Jr. Protective effects of niacin against methylmercury-induced genotoxicity and alterations in antioxidant status in rats. J Toxicol Environ Health A. 2016;79(4):174-83. Epub 2016 Feb 25. PubMed PMID: 26914397.	No CT - no humans
3	El Khoury P, Waldmann E, Huby T, Gall J, Couvert P, Lacorte JM, Chapman J, Frisdal E, Lesnik P, Parhofer KG, Le Goff W, Guerin M. Extended-Release Niacin/Laropiprant Improves Overall Efficacy of Postprandial Reverse Cholesterol Transport. Arterioscler Thromb Vasc Biol. 2016 Feb;36(2):285-94. PubMed PMID: 26681758.	No CT - no humans
4	Ooi EM, Watts GF, Chan DC, Pang J, Tenneti VS, Hamilton SJ, McCormick SP, Marcovina SM, Barrett PH. Effects of extended-release niacin on the postprandial metabolism of Lp(a) and ApoB-100-containing lipoproteins in statin-treated men with type 2 diabetes mellitus. Arterioscler Thromb Vasc Biol. 2015Dec;35(12):2686-93. PubMed PMID: 26515419.	competing risk / special population
5	Croyal M, Ouguerram K, Passard M, Ferchaud-Roucher V, Chétiveaux M, Billon-Crossouard S, de Gouville AC, Lambert G, Krempf M, Nobécourt E. Effects of Extended-Release Nicotinic Acid on Apolipoprotein (a) Kinetics in Hypertriglyceridemic Patients. Arterioscler Thromb Vasc Biol. 2015 Sep;35(9):2042-7. PubMed PMID: 26160958.	No CVD outcomes and follow up shorter than 6 months
6	Gomaraschi M, Ossoli A, Adorni MP, Damonte E, Niesor E, Veglia F, Franceschini G, Benghozi R, Calabresi L. Fenofibrate and extended-release niacin improve the endothelial protective effects of HDL in patients with metabolic syndrome. Vascul Pharmacol. 2015 Nov;74:80-86.	No CVD outcomes and follow up shorter than 6 months
7	Sprecher D, Maxwell M, Goodman J, White B, Tang CM, Boullay V, de Gouville AC. Discovery and characterization of GSK256073, a non-flushing hydroxy-carboxylic acid receptor 2 (HCA2) agonist. Eur J Pharmacol. 2015 Jun 5;756:1-7.	No CT
8	deGoma EM, Salavati A, Shinohara RT, Saboury B, Pollan L, Schoen M, Torigian DA, Mohler ER, Dunbar RL, Litt HI, Woo J, Rader DJ, Alavi A, Mehta NN. A pilot trial to examine the effect of high-dose niacin on arterial wall inflammation using fluorodeoxyglucose positron emission tomography. Acad Radiol. 2015 May;22(5):600-9.	No CVD outcomes and follow up shorter than 6 months
9	Khera AV, Qamar A, Reilly MP, Dunbar RL, Rader DJ. Effects of niacin, statin, and fenofibrate on circulating proprotein convertase subtilisin/kexin type 9 levels in patients with dyslipidemia. Am J Cardiol. 2015 Jan 15;115(2):178-82.	No CVD outcomes and follow up shorter than 6 months
10	Jiang RR, Zhao GP, Zhao JP, Chen JL, Zheng MQ, Liu RR, Wen J. Influence of dietary nicotinic acid supplementation on lipid metabolism and related gene expression in two distinct broiler breeds of female chickens. J Anim Physiol Anim Nutr (Berl). 2014 Oct;98(5):822-9. PubMed PMID: 25356484.	No CT - no humans
11	Hobbs T, Caso R, McMahon D, Nymark M. A novel, multi-ingredient supplement to manage elevated blood lipids in patients with no evidence of cardiovascular disease: a pilot study. Altern Ther Health Med. 2014 Sep-Oct;20(5):18-23.	No niacin - niacin as dietary supplement
12	HPS2-THRIVE Collaborative Group. HPS2-THRIVE randomized placebo-controlled trial in 25673 high-risk patients of ER niacin/laropiprant: trial design, pre-specified muscle and liver outcomes, and reasons for stopping study treatment. Eur Heart J. 2013 May;34(17):1279-91.	No CT
13	Nasser Figueiredo V, Vendrame F, Colontoni BA, Quinaglia T, Roberto Matos-Souza J, Azevedo Moura F, Coelho OR, de Faria EC, Sposito AC. Short-term effects of extended-release niacin with and without the addition of laropiprant on endothelial function in individuals with low HDL-C: a randomized, controlled crossover trial. Clin Ther. 2014 Jun 1;36(6):961-6.	No CVD outcomes and follow up shorter than 6 months
14	Aye MM, Kilpatrick ES, Afolabi P, Wootton SA, Rigby AS, Coady AM, Sandeman DD, Atkin SL. Postprandial effects of long-term niacin/laropiprant use on glucose and lipid metabolism and on cardiovascular risk in patients with polycystic ovary syndrome. Diabetes Obes Metab. 2014 Jun;16(6):545-52. ClinicalTrials.gov Identifier: NCT01118598	No CVD outcomes and follow up shorter than 6 months

© 2019 D’Andrea E et al. *JAMA Network Open.*

n.	References	Reason(s) for exclusion from qualitative and quantitative analysis
15	27: Cenarro A, Puzo J, Ferrando J, Mateo-Gallego R, Bea AM, Calmarza P, Jarauta E, Civeira F. Effect of Nicotinic acid/Laropiprant in the lipoprotein(a) concentration with regard to baseline lipoprotein(a) concentration and LPA genotype. Metabolism. 2014 Mar;63(3):365-71. doi: 10.1016/j.metabol.2013.10.014. Epub 2013 Nov 6. PubMed PMID: 24333007. ClinicalTrials.gov Identifier: NCT01321034	No CVD outcomes and follow up shorter than 6 months
16	31: Franceschini G, Favari E, Calabresi L, Simonelli S, Bondioli A, Adorni MP, Zimetti F, Gomaraschi M, Coutant K, Rossomanno S, Niesor EJ, Bernini F, Benghozi R. Differential effects of fenofibrate and extended-release niacin on high-density lipoprotein particle size distribution and cholesterol efflux capacity in dyslipidemic patients. J Clin Lipidol. 2013 Sep-Oct;7(5):414-22.	No CVD outcomes and follow up shorter than 6 months
17	32: Niesor EJ, Gauthamadasa K, Silva RA, Suchankova G, Kallend D, Gylling H, Asztalos B, Damonte E, Rossomanno S, Abt M, Davidson WS, Benghozi R. Xanthophylls, phytosterols and pre-β1-HDL are differentially affected by fenofibrate and niacin HDL-raising in a cross-over study. Lipids. 2013 Dec;48(12):1185-96.	No CT - no original data
18	40: Lin C, Grandinetti A, Shikuma C, Souza S, Parikh N, Nakamoto B, Kallianpur KJ, Chow D. The effects of extended release niacin on lipoprotein sub-particle concentrations in HIV-infected patients. Hawaii J Med Public Health. 2013 Apr;72(4):123-7.	competing risk / special population
19	42: Gillard BK, Raya JL, Ruiz-Esponda R, Iyer D, Coraza I, Balasubramanyam A, Pownall HJ. Impaired lipoprotein processing in HIV patients on antiretroviral therapy: aberrant high-density lipoprotein lipids, stability, and function. Arterioscler Thromb Vasc Biol. 2013 Jul;33(7):1714-21. ClinicalTrials.gov NCT00246376.	competing risk / special population
20	45: Williams PT, Zhao XQ, Marcovina SM, Brown BG, Krauss RM. Levels of cholesterol in small LDL particles predict atherosclerosis progression and incident CHD in the HDL-Atherosclerosis Treatment Study (HATS). PLoS One. 2013;8(2):e56782. doi: 10.1371/journal.pone.0056782. Epub 2013 Feb 27. ClinicalTrials.gov NCT00000553.	No CT - no original data
21	49: Phan BA, Muñoz L, Shadzi P, Isquith D, Triller M, Brown BG, Zhao XQ. Effects of niacin on glucose levels, coronary stenosis progression, and clinical events in subjects with normal baseline glucose levels (<100 mg/dl): a combined analysis of the Familial Atherosclerosis Treatment Study (FATS), HDL-Atherosclerosis Treatment Study (HATS), Armed Forces Regression Study (AFREGS), and Carotid Plaque Composition by MRI during lipid-lowering (CPC) study. Am J Cardiol. 2013 Feb 1;111(3):352-5.	No CT - no original data
22	47: Keenan JM. Wax-matrix extended-release niacin vs inositol hexanicotinate: a comparison of wax-matrix, extended-release niacin to inositol hexanicotinate "no-flush" niacin in persons with mild to moderate dyslipidemia. J Clin Lipidol. 2013 Jan-Feb;7(1):14-23.	No CVD outcomes and follow up shorter than 6 months
23	50: Dänschel W, Steinhagen-Thiessen E, Buffleben C, Pittrow D, Hildemann SK. Determinants of lipid goal achievement in patients on extended-release nicotinic acid/laropiprant in primary care clinical practice. Curr Med Res Opin. 2013 Jan;29(1):33-40.	No CT
24	51: Hamoud S, Kaplan M, Meilin E, Hassan A, Torgovicky R, Cohen R, Hayek T. Niacin administration significantly reduces oxidative stress in patients with hypercholesterolemia and low levels of high-density lipoprotein cholesterol. Am J Med Sci. 2013 Mar;345(3):195-9. Rambam Health Care Campus, Haifa, Israel (approval number 0258/09).	No outcome of interest - oxidative stress
25	52: Ahmadi F, Shamekhi F, Lessan-Pezeshki M, Khatami MR. Comparison of efficacy of the phosphate binders nicotinic acid and sevelamer hydrochloride in hemodialysis patients. Saudi J Kidney Dis Transpl. 2012 Sep;23(5):934-8. [to discuss]	competing risk / special population
26	56: Karl M, Rubenstein M, Rudnick C, Brejda J. A multicenter study of nutraceutical drinks for cholesterol (evaluating effectiveness and tolerability). J Clin Lipidol. 2012 Mar-Apr;6(2):150-8. ClinicalTrials.gov identifier: NCT01152073	No niacin - niacin as dietary supplement
27	58: Kim SH, Kim MK, Lee HY, Kang HJ, Kim YJ, Park BJ, Kim HS. Efficacy and tolerability of a new extended-release formulation of nicotinic acid in Korean adults with mixed dyslipidemia: an 8-week, multicenter, prospective, randomized, double-blind, and placebo-controlled trial. Clin Ther. 2011 Oct;33(10):1357-64.	No CVD outcomes and follow up shorter than 6 months
28	60: Aramwit P, Srisawadwong R, Supasyndh O. Effectiveness and safety of extended-release nicotinic acid for reducing serum phosphorus in hemodialysis patients. J Nephrol. 2012 May-Jun;25(3):354-62.	competing risk / special population
29	61: Safarova MS, Trukhacheva EP, Ezhov MV, Afanas'eva OI, Afanas'eva MI, Tripoten' MI, Liakishev AA, Pokrovskiĭ SN. [Pleiotropic effects of nicotinic acid therapy in men with coronary heart disease and elevated lipoprotein(a) levels]. Kardiologiia. 2011;51(5):9-16. Russian.	No CVD outcomes

© 2019 D’Andrea E et al. *JAMA Network Open.*

n.	References	Reason(s) for exclusion from qualitative and quantitative analysis
30	Balasubramanyam A, Coraza I, Smith EO, Scott LW, Patel P, Iyer D, Taylor AA, Giordano TP, Sekhar RV, Clark P, Cuevas-Sanchez E, Kamble S, Ballantyne CM, Pownall HJ. Combination of niacin and fenofibrate with lifestyle changes improves dyslipidemia and hypoadiponectinemia in HIV patients on antiretroviral therapy: results of "heart positive," a randomized, controlled trial. J Clin Endocrinol Metab. 2011 Jul;96(7):2236-47. ClinicalTrials.gov NCT00246376.	competing risk / special population
31	Samson SL, Pownall HJ, Scott LW, Ballantyne CM, Smith EO, Sekhar RV, Balasubramanyam A. Heart positive: design of a randomized controlled clinical trial of intensive lifestyle intervention, niacin and fenofibrate for HIV lipodystrophy/dyslipidemia. Contemp Clin Trials. 2006 Dec;27(6):518-30. Epub 2006 Jul 21. ClinicalTrials.gov NCT00246376.	No CT - no original data
32	Mal' GS. [Comparative efficiency of prolonged diet and drug therapies for hyperlipidemias in patients with ischemic heart disease]. Klin Med (Mosk). 2004;82(5):63-6. Russian. PubMed PMID: 15230046.	No CVD outcomes
33	Knab AM, Shanely RA, Henson DA, Jin F, Heinz SA, Austin MD, Nieman DC. Influence of quercetin supplementation on disease risk factors in community-dwelling adults. J Am Diet Assoc. 2011 Apr;111(4):542-9.	No niacin
34	Krasuski RA, Devendra GP, Cater G, Whitney EJ. The effect of gemfibrozil, niacin and cholestyramine combination therapy on metabolic syndrome in the Armed Forces Regression Study. Am J Med Sci. 2011 May;341(5):378-82.	No CT - no original data
35	Dobiásová M, Frohlich J, Sedová M, Cheung MC, Brown BG. Cholesterol esterification and atherogenic index of plasma correlate with lipoprotein size and findings on coronary angiography. J Lipid Res. 2011 Mar;52(3):566-71. (HATS study)	No outcome of interest
36	Salgado BJ, Salgado JV, Dos Santos AM, Casulari LA. Effects of low-dose of niacin associated to simvastatin in the treatment of mixed dyslipidemia Salgad. Minerva Cardioangiol. 2010 Oct;58(5):531-42.	No CVD outcomes and follow up shorter than 6 months
37	Davidson MH, Donovan JM, Misir S, Jones MR. A 50-week extension study on the safety and efficacy of colesevelam in adults with primary hypercholesterolemia. Am J Cardiovasc Drugs. 2010;10(5):305-14.	No niacin
38	Howarth L, Petrisko Y, Furchner-Evanson A, Nemoseck T, Kern M. Snack selection influences nutrient intake, triglycerides, and bowel habits of adult women: a pilot study. J Am Diet Assoc. 2010 Sep;110(9):1322-7.	No CT - pilot study
39	Devendra GP, Whitney EJ, Krasuski RA. Impact of increases in high-density lipoprotein cholesterol on cardiovascular outcomes during the armed forces regression study. J Cardiovasc Pharmacol Ther. 2010 Dec;15(4):380-3.	No CT - no original data
40	Koh Y, Ben-Ezra V, Biggerstaff KD, Nichols DL. Responses of blood lipids and lipoproteins to extended-release niacin and exercise in sedentary postmenopausal women. J Gerontol A Biol Sci Med Sci. 2010 Sep;65(9):924-32.	No CVD outcomes and follow up shorter than 6 months
41	Devine PJ, Turco MA, Taylor AJ. Design and rationale of the ARBITER 6 trial (Arterial Biology for the Investigation of the Treatment Effects of Reducing Cholesterol)-6-HDL and LDL Treatment Strategies in Atherosclerosis (HALTS). Cardiovasc Drugs Ther. 2007 Jun;21(3):221-5. PubMed PMID: 17404825.	No CT
42	Stefanutti C, Mazza F, Vivenzio A, Di Giacomo S, Perrone G, Serra M, Bucci A. Combined treatment with Dif1stat and diet reduce plasma lipid indicators of moderate hypercholesterolemia more effectively than diet alone: a randomized trial in parallel groups. Lipids. 2009 Dec;44(12):1141-8.	No niacin
43	Pan J, Shilian P, Ishida B, Wu X, Kane JP, Malloy MJ, Charles MA. Effect of niacin on preβ-1 high-density lipoprotein levels in diabetes. Metabolism. 2011 Feb;60(2):292-7.	No CT - no original data
44	Chow DC, Stein JH, Seto TB, Mitchell C, Sriratanaviriyakul N, Grandinetti A, Gerschenson M, Shiramizu B, Souza S, Shikuma C. Short-term effects of extended-release niacin on endothelial function in HIV-infected patients on stable antiretroviral therapy. AIDS. 2010 Apr 24;24(7):1019-23.	competing risk / special population
45	Hiatt WR, Hirsch AT, Creager MA, Rajagopalan S, Mohler ER, Ballantyne CM, Regensteiner JG, Treat-Jacobson D, Dale RA, Rooke T. Effect of niacin ER/lovastatin on claudication symptoms in patients with peripheral artery disease. Vasc Med. 2010 Jun;15(3):171-9. doi: 10.1177/1358863X09360579. Mar 8. PubMed PMID: 20212073. ClinicalTrials.gov NCT00062556.	No outcome of interest
47	Kaushik SV, Plaisance EP, Kim T, Huang EY, Mahurin AJ, Grandjean PW, Mathews ST. Extended-release niacin decreases serum fetuin-A concentrations in individuals with metabolic syndrome. Diabetes Metab Res Rev. 2009 Jul;25(5):427-34. doi: 10.1002/dmrr.967.	No outcome of interest

© 2019 D’Andrea E et al. *JAMA Network Open.*

n.	References	Reason(s) for exclusion from qualitative and quantitative analysis
48	Kawata AK, Revicki DA, Thakkar R, Jiang P, Krause S, Davidson MH, Punzi HA, Padley RJ. Flushing ASsessment Tool (FAST): psychometric properties of a new measure assessing flushing symptoms and clinical impact of niacin therapy. Clin Drug Investig. 2009;29(4):215-29.	No outcome of interest
49	Loguercio C, Federico A, Masarone M, Torella R, Blanco Cdel V, Persico M. The impact of diet on liver fibrosis and on response to interferon therapy in patients with HCV-related chronic hepatitis. Am J Gastroenterol. 2008 Dec;103(12):3159-66.	No niacin - niacin as dietary supplement
50	Alves JD, Steinhagen-Thiessen E, Darioli R, Hostalek U, Vogt A. Influence of the timing of low-dose aspirin on tolerability of prolonged-release nicotinic acid in patients at elevated cardiovascular risk. Curr Med Res Opin. 2008 Oct;24(10):2815-20.	No CT - no original data
51	Westphal S, Luley C. Preferential increase in high-molecular weight adiponectin after niacin. Atherosclerosis. 2008 May;198(1):179-83. Epub 2007 Nov 8.	No CT - no original data
52	Ranga GS, Kalra OP, Tandon H, Gambhir JK, Mehrotra G. Effect of aspirin on lipoprotein(a) in patients with ischemic stroke. J Stroke Cerebrovasc Dis. 2007 Sep-Oct;16(5):220-4. PubMed PMID: 17845920.	No CT - no original data
53	Taylor AJ, Zhu D, Sullenberger LE, Lee HJ, Lee JK, Grace KA. Relationship between glycemic status and progression of carotid intima-media thickness during treatment with combined statin and extended-release niacin in ARBITER 2. Vasc Health Risk Manag. 2007;3(1):159-64. [ARBITER 2]	No CT - no original data
54	Norquist JM, Watson DJ, Yu Q, Paolini JF, McQuarrie K, Santanello NC. Validation of a questionnaire to assess niacin-induced cutaneous flushing. Curr Med Res Opin. 2007 Jul;23(7):1549-60. PubMed PMID: 17559750.	No CT - no original data
55	Sharma M, Sharma DR, Singh V, Panwar RB, Hira HS, Mohan B, Kumar N, Sharma SK, Gupta R. Evaluation of efficacy and safety of fixed dose lovastatin and niacin(ER) combination in asian Indian dyslipidemic patients: a multicentric study. Vasc Health Risk Manag. 2006;2(1):87-93.	No CVD outcomes and follow up shorter than 6 months
56	Dubé MP, Wu JW, Aberg JA, Deeg MA, Alston-Smith BL, McGovern ME, Lee D, Shriver SL, Martinez AI, Greenwald M, Stein JH; AIDS Clinical Trials Group A5148 Study Team. Safety and efficacy of extended-release niacin for the treatment of dyslipidaemia in patients with HIV infection: AIDS Clinical Trials Group Study A5148. Antivir Ther. 2006;11(8):1081-9.	No CT - no original data
57	Li XP, Duan J, Zhao SP, Tan MY, Xu ZM, Zhang DQ. [Efficacy and safety of extended-release niacin alone or with atorvastatin for lipid profile modification]. Zhonghua Yi Xue Za Zhi. 2006 Sep 12;86(34):2399-403. Chinese. PubMed PMID: 17156651.	No CVD outcomes and follow up shorter than 6 months
58	Singh U, Otvos J, Dasgupta A, de Lemos JA, Devaraj S, Jialal I. High-dose alpha-tocopherol therapy does not affect HDL subfractions in patients with coronary artery disease on statin therapy. Clin Chem. 2007 Mar;53(3):525-8. Epub 2007 Jan 18.	No niacin
59	Kuvin JT, Dave DM, Sliney KA, Mooney P, Patel AR, Kimmelstiel CD, Karas RH. Effects of extended-release niacin on lipoprotein particle size, distribution, and inflammatory markers in patients with coronary artery disease. Am J Cardiol. 2006 Sep 15;98(6):743-5.	No CT - no original data
60	Gerber MT, Mondy KE, Yarasheski KE, Drechsler H, Claxton S, Stoneman J, DeMarco D, Powderly WG, Tebas P. Niacin in HIV-infected individuals with hyperlipidemia receiving potent antiretroviral therapy. Clin Infect Dis. 2004 Aug 1;39(3):419-25. Epub 2004 Jul 16. PubMed PMID: 15307011.	competing risk / special population
61	Westphal S, Borucki K, Taneva E, Makarova R, Luley C. Extended-release niacin raises adiponectin and leptin. Atherosclerosis. 2007 Aug;193(2):361-5.	No CVD outcomes and follow up shorter than 6 months
62	Glueck CJ, Aregawi D, Agloria M, Khalil Q, Winiarska M, Munjal J, Gogineni S, Wang P. Rosuvastatin 5 and 10 mg/d: a pilot study of the effects in hypercholesterolemic adults unable to tolerate other statins and reach LDL cholesterol goals with nonstatin lipid-lowering therapies. Clin Ther. 2006 Jun;28(6):933-42.	No CT - no original data
63	Chang AM, Smith MJ, Galecki AT, Bloem CJ, Halter JB. Impaired beta-cell function in human aging: response to nicotinic acid-induced insulin resistance. J Clin Endocrinol Metab. 2006 Sep;91(9):3303-9. Epub 2006 Jun 6.	No outcome of interest
64	Zambon A, Brown BG, Hokanson JE, Motulsky AG, Brunzell JD. Genetically determined apo B levels and peak LDL density predict angiographic response to intensive lipid-lowering therapy. J Intern Med. 2006 Apr;259(4):401-9.	No CVD outcomes
65	Vogt A, Kassner U, Hostalek U, Steinhagen-Thiessen E; NAUTILUS Study Group. Evaluation of the safety and tolerability of prolonged-release nicotinic acid in a usual care setting: the NAUTILUS study. Curr Med Res Opin. 2006 Feb;22(2):417-25.	No outcome of interest
66	Canner PL, Furberg CD, McGovern ME. Benefits of niacin in patients with versus without the metabolic syndrome and healed myocardial infarction (from the Coronary Drug Project). Am J Cardiol. 2006 Feb 15;97(4):477-9. Epub 2005 Dec 21.	No CT - no original data

© 2019 D'Andrea E et al. *JAMA Network Open.*

n.	References	Reason(s) for exclusion from qualitative and quantitative analysis
67	Benjó AM, Maranhão RC, Coimbra SR, Andrade AC, Favarato D, Molina MS, Brandizzi LI, da Luz PL. Accumulation of chylomicron remnants and impaired vascular reactivity occur in subjects with isolated low HDL cholesterol: effects of niacin treatment. Atherosclerosis. 2006 Jul;187(1):116-22. Epub 2006 Feb 3.	No outcome of interest
68	Cicero AF, Brancaleoni M, Laghi L, Donati F, Mino M. Antihyperlipidaemic effect of a Monascus purpureus brand dietary supplement on a large sample of subjects at low risk for cardiovascular disease: a pilot study. Complement Ther Med. 2005 Dec;13(4):273-8.	No niacin
69	Hughes TA, Stentz F, Gettys T, Smith SR. Combining beta-adrenergic and peroxisome proliferator-activated receptor gamma stimulation improves lipoprotein composition in healthy moderately obese subjects. Metabolism. 2006 Jan;55(1):26-34.	No niacin
70	Desai MY, Rodriguez A, Wasserman BA, Gerstenblith G, Agarwal S, Kennedy M, Bluemke DA, Lima JA. Association of cholesterol subfractions and carotid lipid core measured by MRI. Arterioscler Thromb Vasc Biol. 2005 Jun;25(6):e110-1	No CT - no original data
71	Burgess JW, Neville TA, Rouillard P, Harder Z, Beanlands DS, Sparks DL. Phosphatidylinositol increases HDL-C levels in humans. J Lipid Res. 2005 Feb;46(2):350-5.	No niacin
72	Preuss HG, Bagchi D, Bagchi M, Rao CV, Dey DK, Satyanarayana S. Effects of a natural extract of (-)-hydroxycitric acid (HCA-SX) and a combination of HCA-SX plus niacin-bound chromium and Gymnema sylvestre extract on weight loss. Diabetes Obes Metab. 2004 May;6(3):171-80.	No outcome of interest
73	Hecht HS, Harman SM. Relation of aggressiveness of lipid-lowering treatment to changes in calcified plaque burden by electron beam tomography. Am J Cardiol. 2003 Aug 1;92(3):334-6.	No CVD outcomes
74	See VY Jr, DeNofrio D, Goldberg L, Chang G, Sasseen B, Kolansky DM, Pickering F, Kao A, Loh E, Wilensky RL. Effect of atorvastatin on postcardiac transplant increase in low-density lipoprotein cholesterol reduces development of intimal hyperplasia and progression of endothelial dysfunction. Am J Cardiol. 2003 Jul 1;92(1):11-5.	competing risk / special population
75	Baskin F, Rosenberg RN, Fang X, Hynan LS, Moore CB, Weiner M, Vega GL. Correlation of statin-increased platelet APP ratios and reduced blood lipids in AD patients. Neurology. 2003 Jun 24;60(12):2006-7.	competing risk / special population
76	Vega GL, Weiner MF, Lipton AM, Von Bergmann K, Lutjohann D, Moore C, Svetlik D. Reduction in levels of 24S-hydroxycholesterol by statin treatment in patients with Alzheimer disease. Arch Neurol. 2003 Apr;60(4):510-5.	competing risk / special population
77	Matthan NR, Giovanni A, Schaefer EJ, Brown BG, Lichtenstein AH. Impact of simvastatin, niacin, and/or antioxidants on cholesterol metabolism in CAD patients with low HDL. J Lipid Res. 2003 Apr;44(4):800-6.	No CT - no original data
78	Fessel WJ, Follansbee SE, Rego J. High-density lipoprotein cholesterol is low in HIV-infected patients with lipodystrophic fat expansions: implications for pathogenesis of fat redistribution. AIDS. 2002 Sep 6;16(13):1785-9.	competing risk / special population
79	Zhao XQ, Yuan C, Hatsukami TS, Frechette EH, Kang XJ, Maravilla KR, Brown BG. Effects of prolonged intensive lipid-lowering therapy on the characteristics of carotid atherosclerotic plaques in vivo by MRI: a case-control study. Arterioscler Thromb Vasc Biol. 2001 Oct;21(10):1623-9.	No CT - no original data
80	Preuss HG, Wallerstedt D, Talpur N, Tutuncuoglu SO, Echard B, Myers A, Bui M, Bagchi D. Effects of niacin-bound chromium and grape seed proanthocyanidin extract on the lipid profile of hypercholesterolemic subjects: a pilot study. J Med. 2000;31(5-6):227-46.	No niacin
81	Sposito AC, Mansur AP, Maranhão RC, Rodrigues-Sobrinho CR, Coelho OR, Ramires JA. Etofibrate but not controlled-release niacin decreases LDL cholesterol and lipoprotein (a) in type IIb dyslipidemic subjects. Braz J Med Biol Res. 2001 Feb;34(2):177-82.	No niacin
82	Zambon A, Deeb SS, Brown BG, Hokanson JE, Brunzell JD. Common hepatic lipase gene promoter variant determines clinical response to intensive lipid-lowering treatment. Circulation. 2001 Feb 13;103(6):792-8.	No outcome of interest
83	Arntz HR, Agrawal R, Wunderlich W, Schnitzer L, Stern R, Fischer F, Schultheiss HP. Beneficial effects of pravastatin (+/-colestyramine/niacin) initiated immediately after a coronary event (the randomized Lipid-Coronary Artery Disease [L-CAD] Study). Am J Cardiol. 2000 Dec 15;86(12):1293-8.	No niacin - niacin not constantly present in the experimental group
84	Garg R, Elam MB, Crouse JR 3rd, Davis KB, Kennedy JW, Egan D, Herd JA, Hunninghake DB, Johnson WC, Kostis JB, Sheps DS, Applegate WB. Effective and safe modification of multiple atherosclerotic risk factors in patients with peripheral arterial disease. Am Heart J. 2000 Nov;140(5):792-803. PubMed PMID: 11054628.	No niacin

© 2019 D'Andrea E et al. *JAMA Network Open.*

n.	References	Reason(s) for exclusion from qualitative and quantitative analysis
85	Mack WJ, Xiang M, Shircore AM, Selzer RH, Hodis HN, Azen SP. Efficacy of two lipid-lowering treatments on quantitative coronary angiographic endpoints. Cardiovasc Drugs Ther. 2000 Aug;14(4):411-8. PubMed PMID: 10999648.	No CT - no original data
86	Mack WJ, LaBree L, Liu C, Selzer RH, Hodis HN. Correlations between measures of atherosclerosis change using carotid ultrasonography and coronary angiography. Atherosclerosis. 2000 Jun;150(2):371-9.	No outcome of interest
87	Mack WJ, Xiang M, Selzer RH, Hodis HN. Serial quantitative coronary angiography and coronary events. Am Heart J. 2000 Jun;139(6):993-9.	No outcome of interest
88	Bittner V, Simon JA, Fong J, Blumenthal RS, Newby K, Stefanick ML. Correlates of high HDL cholesterol among women with coronary heart disease. Am Heart J. 2000 Feb;139(2 Pt 1):288-96.	No CT - no original data
89	Garg R, Malinow M, Pettinger M, Upson B, Hunninghake D. Niacin treatment increases plasma homocyst(e)ine levels. Am Heart J. 1999 Dec;138(6 Pt 1):1082-7.	No outcome of interest
90	Arntz HR, Wunderlich W, Schnitzer L, Stern R, Fischer F, Agrawal R, Linderer T, Schultheiss HP. [Short- and long-term effects of intensified versus conventional antilipidemic therapy in patients with coronary heart disease. Results from the Lipid-Coronary Artery Disease (L-CAD) Study]. Z Kardiol. 1999 Aug;88(8):582-90. German.	No niacin - niacin not constantly present in the experimental group
91	de la Serna G, Cadarso C. Fenofibrate decreases plasma fibrinogen, improves lipid profile, and reduces uricemia. Clin Pharmacol Ther. 1999 Aug;66(2):166-72.	No niacin
92	Kaczmarski M, Wójcicki J, Samochowiec L, Dutkiewicz T, Sych Z. The influence of exogenous antioxidants and physical exercise on some parameters associated with production and removal of free radicals. Pharmazie. 1999 Apr;54(4):303-6.	No outcome of interest
93	Zambon A, Hokanson JE, Brown BG, Brunzell JD. Evidence for a new pathophysiological mechanism for coronary artery disease regression: hepatic lipase-mediated changes in LDL density. Circulation. 1999 Apr 20;99(15):1959-64.	No outcome of interest
94	Hoogerbrugge N. Effects of atorvastatin on serum lipids of patients with familial hypercholesterolaemia. J Intern Med. 1998 Aug;244(2):143-7.	No niacin
95	Ortega RM, Quintas ME, Andrés P, Gaspar MJ, López-Sobaler AM, Navia B, Requejo AM. [Dietary cholesterol as a conditioner of ingestion of other nutrients and various blood parameters in young women]. Nutr Hosp. 1998 Sep-Oct;13(5):221-7. Review. Spanish.	No niacin - niacin as dietary supplement
96	Philipp CS, Cisar LA, Saidi P, Kostis JB. Effect of niacin supplementation on fibrinogen levels in patients with peripheral vascular disease. Am J Cardiol. 1998 Sep 1;82(5):697-9, A9.	No CT - no original data
97	Chojnowska-Jezierska J, Adamska-Dyniewska H. [Prolonged treatment with slow release nicotinic acid in patients with type II hyperlipidemia]. Pol Arch Med Wewn. 1997 Nov;98(11):391-9. Polish.	No CVD outcomes and follow up shorter than 6 months
98	Andrews TC, Whitney EJ, Green G, Kalenian R, Personius BE. Effect of gemfibrozil +/- niacin +/- cholestyramine on endothelial function in patients with serum low-density lipoprotein cholesterol levels <160 mg/dl and high-density lipoprotein cholesterol levels <40 mg/dl. Am J Cardiol. 1997 Oct 1;80(7):831-5.	No niacin
99	Obarzanek E, Hunsberger SA, Van Horn L, Hartmuller VV, Barton BA, Stevens VJ, Kwiterovich PO, Franklin FA, Kimm SY, Lasser NL, Simons-Morton DG, Lauer RM. Safety of a fat-reduced diet: the Dietary Intervention Study in Children (DISC). Pediatrics. 1997 Jul;100(1):51-9.	No niacin - niacin as dietary supplement
100	Johansson JO, Egberg N, Asplund-Carlson A, Carlson LA. Nicotinic acid treatment shifts the fibrinolytic balance favourably and decreases plasma fibrinogen in hypertriglyceridaemic men. J Cardiovasc Risk. 1997 Jun;4(3):165-71.	No CVD outcomes and follow up shorter than 6 months
101	Thomas VL, Gropper SS. Effect of chromium nicotinic acid supplementation on selected cardiovascular disease risk factors. Biol Trace Elem Res. 1996 Dec;55(3):297-305.	No CVD outcomes and follow up shorter than 6 months
102	Azen SP, Qian D, Mack WJ, Sevanian A, Selzer RH, Liu CR, Liu CH, Hodis HN. Effect of supplementary antioxidant vitamin intake on carotid arterial wall intima-media thickness in a controlled clinical trial of cholesterol lowering. Circulation. 1996 Nov 15;94(10):2369-72.	No niacin
103	Schectman G, Wolff N, Byrd JC, Hiatt JG, Hartz A. Physician extenders for cost-effective management of hypercholesterolemia. J Gen Intern Med. 1996 May;11(5):277-86.	No CT
104	Oster G, Borok GM, Menzin J, Heyse JF, Epstein RS, Quinn V, Benson V, Dudl RJ, Epstein AM. Cholesterol-reduction intervention study (CRIS): a randomized trial to assess effectiveness and costs in clinical practice. Arch Intern Med. 1996 Apr 8;156(7):731-9.	No niacin
105	Jokubaitis LA. Fluvastatin in combination with other lipid-lowering agents. Br J Clin Pract Suppl. 1996 Jan;77A:28-32. Review.	No CT - no original data

n.	References	Reason(s) for exclusion from qualitative and quantitative analysis
106	Azen SP, Mack WJ, Cashin-Hemphill L, LaBree L, Shircore AM, Selzer RH, Blankenhorn DH, Hodis HN. Progression of coronary artery disease predicts clinical coronary events. Long-term follow-up from the Cholesterol Lowering Atherosclerosis Study. Circulation. 1996 Jan 1;93(1):34-41.	No niacin
107	Maher VM, Brown BG, Marcovina SM, Hillger LA, Zhao XQ, Albers JJ. Effects of lowering elevated LDL cholesterol on the cardiovascular risk of lipoprotein(a). JAMA. 1995 Dec 13;274(22):1771-4.	No niacin
108	Schiel R, Bambauer R, Müller UA. Four years' treatment efficacy of patients with severe hyperlipidemia. Lipid lowering drugs versus LDL-apheresis. Int J Artif Organs. 1995 Dec;18(12):786-93.	No niacin
109	Jensen RA, Lal SM, Diaz-Arias A, James-Kracke M, Van Stone JC, Ross G Jr. Does cholestyramine interfere with cyclosporine absorption? A prospective study in renal transplant patients. ASAIO J. 1995 Jul-Sep;41(3):M704-6.	No niacin
110	Hodis HN, Mack WJ, LaBree L, Cashin-Hemphill L, Sevanian A, Johnson R, Azen SP. Serial coronary angiographic evidence that antioxidant vitamin intake reduces progression of coronary artery atherosclerosis. JAMA. 1995 Jun 21;273(23):1849-54.	No CT - no original data
111	Janelle KC, Barr SI. Nutrient intakes and eating behavior scores of vegetarian and nonvegetarian women. J Am Diet Assoc. 1995 Feb;95(2):180-6, 189, quiz 187-8.	No niacin
112	Oster G, Borok GM, Menzin J, Heys JF, Epstein RS, Quinn V, Benson VV, Dudl RJ, Epstein A. A randomized trial to assess effectiveness and cost in clinical practice: rationale and design of the Cholesterol Reduction Intervention Study (CRIS). Control Clin Trials. 1995 Feb;16(1):3-16.	No CT - no original data
113	Hodis HN. Reversibility of atherosclerosis--evolving perspectives from two arterial imaging clinical trials: the cholesterol lowering atherosclerosis regression study and the monitored atherosclerosis regression study. J Cardiovasc Pharmacol. 1995;25 Suppl 4:S25-31.	No CT - no original data
114	Jokubaitis LA. Fluvastatin in combination with other lipid-lowering agents. Br J Clin Pract Suppl. 1994 Dec;(77):28-32. Update in: Br J Clin Pract Suppl. 1996 Jan;77A:28-32.	No CT - no original data
115	Jacobson TA, Jokubaitis LA, Amorosa LF. Fluvastatin and niacin in hypercholesterolemia: a preliminary report on gender differences in efficacy. Am J Med. 1994 Jun 6;96(6A):64S-68S.	No CT
116	Daida H, Lee YJ, Yokoi H, Kanoh T, Ishiwata S, Kato K, Nishikawa H, Takatsu F, Kato H, Kutsumi Y, et al. Prevention of restenosis after percutaneous transluminal coronary angioplasty by reducing lipoprotein (a) levels with low-density lipoprotein apheresis. Low-Density Lipoprotein Apheresis Angioplasty Restenosis Trial (L-ART) Group. Am J Cardiol. 1994 Jun 1;73(15):1037-40.	No niacin
117	Jacobson TA, Amorosa LF. Combination therapy with fluvastatin and niacin in hypercholesterolemia: a preliminary report on safety. Am J Cardiol. 1994 May 26;73(14):25D-29D.	No outcome of interest
118	Stewart BF, Brown BG, Zhao XQ, Hillger LA, Sniderman AD, Dowdy A, Fisher LD, Albers JJ. Benefits of lipid-lowering therapy in men with elevated apolipoprotein B are not confined to those with very high low density lipoprotein cholesterol. J Am Coll Cardiol. 1994 Mar 15;23(4):899-906.	No CT - no original data
119	Davignon J, Roederer G, Montigny M, Hayden MR, Tan MH, Connelly PW, Hegele R, McPherson R, Lupien PJ, Gagné C, et al. Comparative efficacy and safety of pravastatin, nicotinic acid and the two combined in patients with hypercholesterolemia. Am J Cardiol. 1994 Feb 15;73(5):339-45.	No CVD outcomes and follow up shorter than 6 months
120	Kiseleva NG. [Induracin, prolonged-action nicotinic acid, in the treatment of atherogenic dyslipoproteinemia]. Klin Med (Mosk). 1994;72(3):20-4. Russian.	No CT - no original data
121	Zhao XQ, Brown BG, Hillger L, Sacco D, Bisson B, Fisher L, Albers JJ.Effects of intensive lipid-lowering therapy on the coronary arteries of asymptomatic subjects with elevated apolipoprotein B. Circulation. 1993 Dec;88(6):2744-53.	No CT - no original data
122	Mack WJ, Selzer RH, Hodis HN, Erickson JK, Liu CR, Liu CH, Crawford DW, Blankenhorn DH. One-year reduction and longitudinal analysis of carotid intima-media thickness associated with colestipol/niacin therapy. Stroke. 1993 Dec;24(12):1779-83.	No CT - no original data
123	Smith HT, Jokubaitis LA, Troendle AJ, Hwang DS, Robinson WT. Pharmacokinetics of fluvastatin and specific drug interactions. Am J Hypertens. 1993 Nov;6(11 Pt 2):375S-382S.	No niacin
124	Blankenhorn DH, Selzer RH, Crawford DW, Barth JD, Liu CR, Liu CH, Mack WJ, Alaupovic P. Beneficial effects of colestipol-niacin therapy on the common carotid artery. Two- and four-year reduction of intima-media thickness measured by ultrasound. Circulation. 1993 Jul;88(1):20-8.	No CT - no original data

© 2019 D’Andrea E et al. *JAMA Network Open.*

n.	References	Reason(s) for exclusion from qualitative and quantitative analysis
125	Patterson MC, Di Bisceglie AM, Higgins JJ, Abel RB, Schiffmann R, Parker CC, Argoff CE, Grewal RP, Yu K, Pentchev PG, et al. The effect of cholesterol-lowering agents on hepatic and plasma cholesterol in Niemann-Pick disease type C. Neurology. 1993 Jan;43(1):61-4.	competing risk / special population
126	Oganov RG, Kiseleva NG, Aronov DM, Akhmedzhanov NM, Olfer'ev AM, Kiseleva NS, Perova NV. [The use of Enduracin--a prolonged-action form of nicotinic acid--in correcting atherogenic dyslipidemias]. Kardiologiia. 1993;33(10):54-9, 6. Russian.	No CVD outcomes
127	Grundy SM. Cholesterol-lowering drugs as cardioprotective agents. Am J Cardiol. 1992 Dec 21;70(21):27I-32I. Review. PubMed PMID: 1471602.	No CT
128	Squires RW, Allison TG, Gau GT, Miller TD, Kottke BA. Low-dose, time-release nicotinic acid: effects in selected patients with low concentrations of high-density lipoprotein cholesterol. Mayo Clin Proc. 1992 Sep;67(9):855-60.	No CT - no original data
129	Brown BG. Effect of lovastatin or niacin combined with colestipol and regression of coronary atherosclerosis. Eur Heart J. 1992 Jul;13 Suppl B:17-20.	No CT
130	Mack WJ, Selzer RH, Pogoda JM, Lee PL, Shircore AM, Azen SP, Blankenhorn DH. Comparison of computer- and human-derived coronary angiographic end-point measures for controlled therapy trials. Arterioscler Thromb. 1992 Mar;12(3):348-56. PubMed PMID: 1547194.	No outcome of interest
131	Whelan AM, Price SO, Fowler SF, Hainer BL. The effect of aspirin on niacin-induced cutaneous reactions. J Fam Pract. 1992 Feb;34(2):165-8.	No outcome of interest
132	Keenan JM, Bae CY, Fontaine PL, Wenz JB, Myers S, Huang ZQ, Ripsin C. Treatment of hypercholesterolemia: comparison of younger versus older patients using wax-matrix sustained-release niacin. J Am Geriatr Soc. 1992 Jan;40(1):12-8.	No niacin
133	Tornvall P, Walldius G. A comparison between nicotinic acid and acipimox in hypertriglyceridaemia--effects on serum lipids, lipoproteins, glucose tolerance and tolerability. J Intern Med. 1991 Nov;230(5):415-21.	No CVD outcomes and follow up shorter than 6 months
134	Mack WJ, Blankenhorn DH. Factors influencing the formation of new human coronary lesions: age, blood pressure, and blood cholesterol. Am J Public Health. 1991 Sep;81(9):1180-4.	No CT - no original data
135	Azen SP, Cashin-Hemphill L, Pogoda J, Mack WJ, Sanmarco ME, Wickham E, Blankenhorn DH. Evaluation of human panelists in assessing coronary atherosclerosis. Arterioscler Thromb. 1991 Mar-Apr;11(2):385-94.	No CT - no original data
136	Blankenhorn DH, Azen SP, Crawford DW, Nessim SA, Sanmarco ME, Selzer RH, Shircore AM, Wickham EC. Effects of colestipol-niacin therapy on human femoral atherosclerosis. Circulation. 1991 Feb;83(2):438-47. PubMed PMID: 1991366.	No CT - no original data
137	Cashin-Hemphill L, Mack WJ, Pogoda JM, Sanmarco ME, Azen SP, Blankenhorn DH. Beneficial effects of colestipol-niacin on coronary atherosclerosis. A 4-year follow-up. JAMA. 1990 Dec 19;264(23):3013-7.	No CT - no original data
138	Garg A, Grundy SM. Nicotinic acid as therapy for dyslipidemia in non-insulin-dependent diabetes mellitus. JAMA. 1990 Aug 8;264(6):723-6.	competing risk / special population
139	Blankenhorn DH, Alaupovic P, Wickham E, Chin HP, Azen SP. Prediction of angiographic change in native human coronary arteries and aortocoronary bypass grafts. Lipid and nonlipid factors. Circulation. 1990 Feb;81(2):470-6.	No CT - no original data
140	Kuo PT. Management of blood lipid abnormalities in coronary heart disease patients. Clin Cardiol. 1989 Oct;12(10):553-60. Review.	No CT
141	Secondary prevention of coronary disease with lipid-lowering drugs. Lancet. 1989 Mar 4;1(8636):473-4. Review.	No CT
142	Mantell G. Lipid lowering drugs in atherosclerosis--the HMG-CoA reductase inhibitors. Clin Exp Hypertens A. 1989;11(5-6):927-41. Review.	No CT
143	Tobert JA. Efficacy and long-term adverse effect pattern of lovastatin. Am J Cardiol. 1988 Nov 11;62(15):28J-34J. Review.	No CT
144	Bilheimer DW. Therapeutic control of hyperlipidemia in the prevention of coronary atherosclerosis: a review of results from recent clinical trials. Am J Cardiol. 1988 Nov 11;62(15):1J-9J. Review.	No CT
145	Lavie CJ, Gau GT, Squires RW, Kottke BA. Management of lipids in primary and secondary prevention of cardiovascular diseases. Mayo Clin Proc. 1988 Jun;63(6):605-21. Review.	No CT

© 2019 D'Andrea E et al. *JAMA Network Open.*

n.	References	Reason(s) for exclusion from qualitative and quantitative analysis
146	Guarini G, Costa C, Trimarco C, Santese D, Vitaliano E. [Effects on lipid metabolism of inhibitors of cholesterol synthesis]. Clin Ter. 1988 Apr 15;125(1):41-50. Italian.	No niacin
147	Blankenhorn DH, Johnson RL, Nessim SA, Azen SP, Sanmarco ME, Selzer RH. The Cholesterol Lowering Atherosclerosis Study (CLAS): design, methods, and baseline results. Control Clin Trials. 1987 Dec;8(4):356-87.	No CT - no original data
148	Hoeg JM, Maher MB, Bailey KR, Zech LA, Gregg RE, Sprecher DL, Brewer HB Jr. Effects of combination cholestyramine-neomycin treatment on plasma lipoprotein concentrations in type II hyperlipoproteinemia. Am J Cardiol. 1985 May 1;55(11):1282-6.	No niacin
149	Odetti P, Cheli V, Carta G, Maiello M, Viviani GL. Effect of nicotinic acid associated with retinol and tocopherols on plasma lipids in hyperlipoproteinaemic patients. Pharmatherapeutica. 1984;4(1):21-4.	No niacin
150	Steger W. [Therapy of lipoproteinemia. Comparison between Lipo-Merz retard and a commercial combination drug]. Fortschr Med. 1980 May 15;98(18):(718-20).German.	No English Italian or Spanish
151	Brinton EA, Triscari J, Brudi P, Chen E, Johnson-Levonas AO, Sisk CM, Ruck RA, MacLean AA, Maccubbin D, Mitchel YB. Effects of extended-release niacin/laropiprant on correlations between apolipoprotein B, LDL-cholesterol and non-HDL-cholesterol in patients with type 2 diabetes. Lipids Health Dis. 2016 Jul 12;15(1):116. PubMed PMID: 27405296; Protocol 069, Clinical Trials.gov: NCT00485758	No CT - no original data
152	Bays HE, Brinton EA, Triscari J, Chen E, Maccubbin D, MacLean AA, Gibson KL, Ruck RA, Johnson-Levonas AO, O'Neill EA, Mitchel YB. Extended-release niacin/laropiprant significantly improves lipid levels in type 2 diabetes mellitus irrespective of baseline glycemic control. Vasc Health Risk Manag. 2015 Feb 24;11:165-72. ClinicalTrials.gov Identifier: NCT00485758	No CT - no original data
153	Bays H, Giezek H, McKenney JM, O'Neill EA, Tershakovec AM. Extended-release niacin/laropiprant effects on lipoprotein subfractions in patients with type 2 diabetes mellitus. Metab Syndr Relat Disord. 2012 Aug;10(4):260-6. ClinicalTrials.gov Identifier: NCT00485758	competing risk / special population
154	Bays H, Shah A, Dong Q, McCrary Sisk C, Maccubbin D. Extended-release niacin/laropiprant lipid-altering consistency across patient subgroups. Int J Clin Pract. 2011 Apr;65(4):436-45. ClinicalTrials.gov Identifier: NCT00485758, NCT00479388, NCT00269217, NCT00269204	No CT - no original data
155	Ballantyne C, Gleim G, Liu N, Sisk CM, Johnson-Levonas AO, Mitchel Y. Effects of coadministered extended-release niacin/laropiprant and simvastatin on lipoprotein subclasses in patients with dyslipidemia. J Clin Lipidol. 2012 May-Jun;6(3):235-43. doi: 10.1016/j.jacl.2011.11.004. Epub 2011 Dec 3. ClinicalTrials.gov Identifier: NCT00269217	No CT - no original data
156	Shah S, Ceska R, Gil-Extremera B, Paolini JF, Giezek H, Vandormael K, Mao A, McCrary Sisk C, Maccubbin D. Efficacy and safety of extended-release niacin/laropiprant plus statin vs. doubling the dose of statin in patients withprimary hypercholesterolaemia or mixed dyslipidaemia. Int J Clin Pract. 2010 May;64(6):727-38. ClinicalTrials.gov Identifier: NCT00479388	No CVD outcomes and follow up shorter than 6 months
157	Farnier M, Chen E, Johnson-Levonas AO, McCrary Sisk C, Mitchel YB. Effects of extended-release niacin/laropiprant, simvastatin, and the combination on correlations between apolipoprotein B, LDL cholesterol, and non-HDL cholesterol in patients with dyslipidemia. Vasc Health Risk Manag. 2014 May 7;10:279-90. ClinicalTrials.gov Identifier: NCT00269217	No CT - no original data
158	Yadav R, Liu Y, Kwok S, Hama S, France M, Eatough R, Pemberton P, Schofield J, Siahmansur TJ, Malik R, Ammori BA, Issa B, Younis N, Donn R, Stevens A, Durrington P, Soran H. Effect of Extended-Release Niacin on High-Density Lipoprotein (HDL) Functionality, Lipoprotein Metabolism, and Mediators of Vascular Inflammation in Statin-Treated Patients. J Am Heart Assoc. 2015 Sep 15;4(9):e001508. doi: 10.1161/JAHA.114.001508. PubMed PMID: 26374297; PubMed Clinical Trials.gov: NCT01054508.	No CVD outcomes and follow up shorter than 6 months
159	12: Savinova OV, Fillaus K, Harris WS, Shearer GC. Effects of niacin and omega-3 fatty acids on the apolipoproteins in overweight patients with elevated triglycerides and reduced HDL cholesterol. Atherosclerosis. 2015 Jun;240(2):520-5. PubMed PMID: 25932792. ClinicalTrials.gov NCT00286234.	No CT - no original data
160	Hu S, Shearer GC, Steffes MW, Harris WS, Bostom AG. Once-daily extended-release niacin lowers serum phosphorus concentrations in patients with metabolic syndrome dyslipidemia. Am J Kidney Dis. 2011 Jan;57(1):181-2. doi: 10.1053/j.ajkd.2010.06.029. Epub 2010 Oct 8. [EMBASE] ClinicalTrials.gov NCT00286234	No CVD outcomes and follow up shorter than 6 months

© 2019 D'Andrea E et al. *JAMA Network Open.*

n.	References	Reason(s) for exclusion from qualitative and quantitative analysis
161	Shearer GC, Pottala JV, Hansen SN, Brandenburg V, Harris WS. Effects of prescription niacin and omega-3 fatty acids on lipids and vascular function in metabolic syndrome: a randomized controlled trial. J Lipid Res. 2012 Nov;53(11):2429-35. doi: 10.1194/jlr.P022392. Epub 2012 Aug 14. PubMed PMID: 22892157; PubMed Central PMCID: PMC3466011. ClinicalTrials.gov NCT00286234.	No CVD outcomes and follow up shorter than 6 months
162	Guerra A, Rangan BV, Coleman A, Xu H, Kotsia A, Christopoulos G, Sosa A, Chao H, Han H, Abdurrahim G, Roesle M, de Lemos JA, McGuire DK, Packer M, Banerjee S, Brilakis ES. Effect of Extended-Release Niacin on Carotid Intima Media Thickness, Reactive Hyperemia, and Endothelial Progenitor Cell Mobilization: Insights From the Atherosclerosis Lesion Progression Intervention Using Niacin Extended Release in Saphenous Vein Grafts (ALPINE-SVG) Pilot Trial. J Invasive Cardiol. 2015	No outcome of interest
163	Yasmeen G, Dawani ML, Mahboob T. Association of high-density lipoprotein cholesterol with improvement of endothelial dysfunction recovery in renovascular disease. Iran J Kidney Dis. 2015 Jan;9(1):39-45. PubMed PMID: 25599735.	competing risk / special population
164	Masana L, Cabré A, Heras M, Amigó N, Correig X, Martínez-Hervás S, Real JT, Ascaso JF, Quesada H, Julve J, Palomer X, Vázquez-Carrera M, Girona J, Plana N, Blanco-Vaca F. Remarkable quantitative and qualitative differences in HDL after niacin or fenofibrate therapy in type 2 diabetic patients. Atherosclerosis. 2015 Feb;238(2):213-9.	competing risk / special population
165	Kalil RS, Wang JH, de Boer IH, Mathew RO, Ix JH, Asif A, Shi X, Boden WE. Effect of extended-release niacin on cardiovascular events and kidney function in chronic kidney disease: a post hoc analysis of the AIM-HIGH trial. Kidney Int. 2015 Jun;87(6):1250-7.	No CT - no original data
166	Albers JJ, Slee A, O'Brien KD, Robinson JG, Kashyap ML, Kwiterovich PO Jr, Xu P, Marcovina SM. Relationship of apolipoproteins A-1 and B, and lipoprotein(a) to cardiovascular outcomes: the AIM-HIGH trial (Atherothrombosis Intervention in Metabolic Syndrome with Low HDL/High Triglyceride and Impact on Global Health Outcomes). J Am Coll Cardiol. 2013 Oct 22;62(17):1575-9.	No CT - no original data
167	Guyton JR, Slee AE, Anderson T, Fleg JL, Goldberg RB, Kashyap ML, Marcovina SM, Nash SD, O'Brien KD, Weintraub WS, Xu P, Zhao XQ, Boden WE. Relationship of lipoproteins to cardiovascular events: the AIM-HIGH Trial (Atherothrombosis Intervention in Metabolic Syndrome With Low HDL/High Triglycerides and Impact on Global Health Outcomes). J Am Coll Cardiol. 2013 Oct 22;62(17):1580-4.	No CT - no original data
168	Teo KK, Goldstein LB, Chaitman BR, Grant S, Weintraub WS, Anderson DC, Sila CA, Cruz-Flores S, Padley RJ, Kostuk WJ, Boden WE; AIM-HIGH Investigators. Extended-release niacin therapy and risk of ischemic stroke in patients with cardiovascular disease: the Atherothrombosis Intervention in Metabolic Syndrome with low HDL/High Triglycerides: Impact on Global Health Outcome (AIM-HIGH) trial. Stroke. 2013 Oct;44(10):2688-93.	No CT - no original data
169	AIM-HIGH Investigators. The role of niacin in raising high-density lipoprotein cholesterol to reduce cardiovascular events in patients with atherosclerotic cardiovascular disease and optimally treated low-density lipoprotein cholesterol: baseline characteristics of study participants. The Atherothrombosis Intervention in Metabolic syndrome with low HDL/high triglycerides: impact on Global Health outcomes (AIM-HIGH) trial. Am Heart J. 2011 Mar;161(3):538-43.	No CT - no original data
170	AIM-HIGH Investigators. The role of niacin in raising high-density lipoprotein cholesterol to reduce cardiovascular events in patients with atherosclerotic cardiovascular disease and optimally treated low-density lipoprotein cholesterol Rationale and study design. The Atherothrombosis Intervention in Metabolic syndrome with low HDL/high triglycerides: Impact on Global Health outcomes (AIM-HIGH). Am Heart J. 2011 Mar;161(3):471-477.e2.	No CT - no original data
171	Kumar S, Rai H, Kapoor A, Tewari S, Sinha N. Pharmacological measures to increase HDL-C among high risk isolated low HDL cases: a randomized study amongst north Indians. Indian J Med Res. 2013 Dec;138(6):873-81. CTRI No. CTRI/2012/003198	No CVD outcomes and follow up shorter than 6 months
172	Pang J, Chan DC, Hamilton SJ, Tenneti VS, Watts GF, Barrett PH. Effect of niacin on high-density lipoprotein apolipoprotein A-I kinetics in statin-treated patients with type 2 diabetes mellitus. Arterioscler Thromb Vasc Biol. 2014 Feb;34(2):427-32. Australian New Zealand Clinical Trials Registry (ACTRN12609000448246).	competing risk / special population
173	Davidson MH, Rooney M, Pollock E, Drucker J, Choy Y. Effect of colesevelam and niacin on low-density lipoprotein cholesterol and glycemic control in subjects with dyslipidemia and impaired fasting glucose. J Clin Lipidol. 2013 Sep-Oct;7(5):423-32.	No CVD outcomes and follow up shorter than 6 months

© 2019 D’Andrea E et al. *JAMA Network Open.*

n.	References	Reason(s) for exclusion from qualitative and quantitative analysis
174	Scoffone HM, Krajewski M, Zorca S, Bereal-Williams C, Littel P, Seamon C, Mendelsohn L, Footman E, Abi-Jaoudeh N, Sachdev V, Machado RF, Cuttica M, Shamburek R, Cannon RO 3rd, Remaley A, Minniti CP, Kato GJ. Effect of extended-release niacin on serum lipids and on endothelial function in adults with sickle cell anemia and low high-density lipoprotein cholesterol levels. Am J Cardiol. 2013 Nov 1;112(9):1499-504. ClinicalTrials.gov identifier NCT00508989	competing risk / special population
175	Kei A, Liberopoulos E, Tellis K, Rizzo M, Elisaf M, Tselepis A. Effect of hypolipidemic treatment on emerging risk factors in mixed dyslipidemia: a randomized pilot trial. Eur J Clin Invest. 2013 Jul;43(7):698-707. ClinicalTrials.gov (NCT01010516).	No CT - no original data
176	Kei A, Liberopoulos E, Tellis C, Elisaf M, Tselepis A. Lipid-modulating treatments for mixed dyslipidemia increase HDL-associated phospholipase A2 activity with differential effects on HDL subfractions. Lipids. 2013 Oct;48(10):957-65. ClinicalTrials.gov (NCT01010516).	No CT - no original data
177	Kei A, Liberopoulos EN, Mikhailidis DP, Elisaf M. Comparison of switch to the highest dose of rosuvastatin vs. add-on nicotinic acid vs. add-on fenofibrate for mixed dyslipidaemia. Int J Clin Pract. 2013 May;67(5):412-9. ClinicalTrials.gov (NCT01010516).	No CVD outcomes and follow up shorter than 6 months
178	Le NA, Jin R, Tomassini JE, Tershakovec AM, Neff DR, Wilson PW. Changes in lipoprotein particle number with ezetimibe/simvastatin coadministered with extended-release niacin in hyperlipidemic patients. J Am Heart Assoc. 2013 Aug 7;2(4):e000037. Clinicaltrials.gov Identifier: NCT00271817	No CT - no original data
179	Fazio S, Guyton JR, Lin J, Tomassini JE, Shah A, Tershakovec AM. Long-term efficacy and safety of ezetimibe/simvastatin coadministered with extended-release niacin in hyperlipidaemic patients with diabetes or metabolic syndrome. Diabetes Obes Metab. 2010 Nov;12(11):983-93. Clinicaltrials.gov Identifier: NCT00271817	competing risk / special population
180	Fazio S, Guyton JR, Polis AB, Adewale AJ, Tomassini JE, Ryan NW, Tershakovec AM. Long-term safety and efficacy of triple combination ezetimibe/simvastatin plus extended-release niacin in patients with hyperlipidemia. Am J Cardiol. 2010 Feb 15;105(4):487-94. Clinicaltrials.gov Identifier: NCT00271817	No CT - no original data
182	Philpott AC, Hubacek J, Sun YC, Hillard D, Anderson TJ. Niacin improves lipid profile but not endothelial function in patients with coronary artery disease on high dose statin therapy. Atherosclerosis. 2013 Feb;226(2):453-8. ClinicalTrials.gov Identifier: NCT00150722	No CVD outcomes and follow up shorter than 6 months
183	Wi J, Kim JY, Park S, Kang SM, Jang Y, Chung N, Shim WH, Cho SY, Lee SH. Optimal pharmacologic approach to patients with hypertriglyceridemia and low high-density lipoprotein-cholesterol: randomized comparison of fenofibrate 160 mg and niacin 1500 mg. Atherosclerosis. 2010 Nov;213(1):235-40. ClinicalTrials.gov number, NCT01122355	No CVD outcomes and follow up shorter than 6 months
184	Hamilton SJ, Chew GT, Davis TM, Watts GF. Niacin improves small artery vasodilatory function and compliance in statin-treated type 2 diabetic patients. Diab Vasc Dis Res. 2010 Oct;7(4):296-9.	competing risk / special population
185	Villines TC, Stanek EJ, Devine PJ, Turco M, Miller M, Weissman NJ, Griffen L, Taylor AJ. The ARBITER 6-HALTS Trial (Arterial Biology for the Investigation of the Treatment Effects of Reducing Cholesterol 6-HDL and LDL Treatment Strategies in Atherosclerosis): final results and the impact of medication adherence, dose, and treatment duration. J Am Coll Cardiol. 2010 Jun 15;55(24):2721-6. ClinicalTrials.gov Identifier: NCT00397657	No CT - no original data
186	Superko HR, Garrett BC, King SB 3rd, Momary KM, Chronos NA, Wood PD. Effect of combination nicotinic acid and gemfibrozil treatment on intermediate density lipoprotein, and subclasses of low density lipoprotein and high density lipoprotein in patients with combined hyperlipidemia. Am J Cardiol. 2009 Feb 1;103(3):387-92.	No CVD outcomes and follow up shorter than 6 months
187	Linke A, Sonnabend M, Fasshauer M, Höllriegel R, Schuler G, Niebauer J, Stumvoll M, Blüher M. Effects of extended-release niacin on lipid profile and adipocyte biology in patients with impaired glucose tolerance. Atherosclerosis. 2009 Jul;205(1):207-13.	competing risk / special population
188	Warnholtz A, Wild P, Ostad MA, Elsner V, Stieber F, Schinzel R, Walter U, Peetz D, Lackner K, Blankenberg S, Munzel T. Effects of oral niacin on endothelial dysfunction in patients with coronary artery disease: results of the randomized, double-blind, placebo-controlled INEF study. Atherosclerosis. 2009 May;204(1):216-21. ISRCT-number 48365122 (http://www.controlled-trials.com/ISRCTN48365122).	No CVD outcomes and follow up shorter than 6 months

© 2019 D’Andrea E et al. *JAMA Network Open.*

n.	References	Reason(s) for exclusion from qualitative and quantitative analysis
189	Lamon-Fava S, Diffenderfer MR, Barrett PH, Buchsbaum A, Nyaku M, Horvath KV, Asztalos BF, Otokozawa S, Ai M, Matthan NR, Lichtenstein AH, Dolnikowski GG, Schaefer EJ. Extended-release niacin alters the metabolism of plasma apolipoprotein (Apo) A-I and ApoB-containing lipoproteins. Arterioscler Thromb Vasc Biol. 2008 Sep;28(9):1672-8.	No CVD outcomes and follow up shorter than 6 months
190	McKenney JM, Jones PH, Bays HE, Knopp RH, Kashyap ML, Ruoff GE, McGovern ME. Comparative effects on lipid levels of combination therapy with a statin and extended-release niacin or ezetimibe versus a statin alone (the COMPELL study). Atherosclerosis. 2007 Jun;192(2):432-7. ClinicalTrials.gov Identifier: NCT00079638	No CVD outcomes and follow up shorter than 6 months
191	Taylor AJ, Lee HJ, Sullenberger LE. The effect of 24 months of combination statin and extended-release niacin on carotid intima-media thickness: ARBITER 3. Curr Med Res Opin. 2006 Nov;22(11):2243-50.	No CT - no original data
192	Superko HR, McGovern ME, Raul E, Garrett B. Differential effect of two nicotinic acid preparations on low-density lipoprotein subclass distribution in patients classified as low-density lipoprotein pattern A, B, or I. Am J Cardiol. 2004 Sep 1;94(5):588-94.	No CVD outcomes and follow up shorter than 6 months
193	Lin TH, Voon WC, Yen HW, Huang CH, Su HM, Lai WT, Sheu SH. Randomized comparative study of the effects of treatment with once-daily, niacin extended-release/lovastatin and with simvastatin on lipid profile and fibrinolytic parameters in Taiwan. Kaohsiung J Med Sci. 2006 Jun;22(6):257-65.	No CVD outcomes and follow up shorter than 6 months
194	Ronsein GE, Hutchins PM, Isquith D, Vaisar T, Zhao XQ, Heinecke JW. Niacin Therapy Increases High-Density Lipoprotein Particles and Total Cholesterol Efflux Capacity But Not ABCA1-Specific Cholesterol Efflux in Statin-Treated Subjects. Arterioscler Thromb Vasc Biol. 2016 Feb;36(2):404-11.	No CT - no original data
195	Bays HE, Shah A, Lin J, McCrary Sisk C, Paolini JF, Maccubbin D. Efficacy and tolerability of extended-release niacin/laropiprant in dyslipidemic patients with metabolic syndrome. J Clin Lipidol. 2010 Nov-Dec;4(6):515-21. ClinicalTrials.gov Identifier: NCT00269204, NCT00479388	No CT - no original data
196	Nash MS, Lewis JE, Dyson-Hudson TA, Szlachcic Y, Yee F, Mendez AJ, Spungen AM, Bauman WA. Safety, tolerance, and efficacy of extended-release niacin monotherapy for treating dyslipidemia risks in persons with chronic tetraplegia: a randomized multicenter controlled trial. Arch Phys Med Rehabil. 2011 Mar;92(3):399-410.	competing risk / special population
197	Stamler J. The coronary drug project--findings with regard to estrogen, dextrothyroxine, clofibrate and niacin. Adv Exp Med Biol. 1977;82:52-75. PubMed PMID: 335823.	No CT - no original data
198	Insull W Jr, McGovern ME, Schrott H, Thompson P, Crouse JR, Zieve F, Corbelli J. Efficacy of extended-release niacin with lovastatin for hypercholesterolemia: assessing all reasonable doses with innovative surface graph analysis. Arch Intern Med. 2004 May 24;164(10):1121-7.	No CVD outcomes and follow up shorter than 6 months
199	Morgan JM, Capuzzi DM, Baksh RI, Intenzo C, Carey CM, Reese D, Walker K. Effects of extended-release niacin on lipoprotein subclass distribution. Am J Cardiol. 2003 Jun 15;91(12):1432-6. PubMed PMID: 12804729.	No CVD outcomes and follow up shorter than 6 months
200	Berge KG, Canner PL. Coronary drug project: experience with niacin. Coronary Drug Project Research Group. Eur J Clin Pharmacol. 1991;40 Suppl 1:S49-51. PubMed PMID: 2044644.	No CT - no original data
201	Bays HE, Dujovne CA, McGovern ME, White TE, Kashyap ML, Hutcheson AG, Crouse JR; ADvicor Versus Other Cholesterol-Modulating Agents Trial Evaluation. Comparison of once-daily, niacin extended-release/lovastatin with standard doses of atorvastatin and simvastatin (the ADvicor Versus Other Cholesterol-Modulating Agents Trial Evaluation [ADVOCATE]). Am J Cardiol. 2003 Mar 15;91(6):667-72.	No CVD outcomes and follow up shorter than 6 months
202	Grundy SM, Vega GL, McGovern ME, Tulloch BR, Kendall DM, Fitz-Patrick D, Ganda OP, Rosenson RS, Buse JB, Robertson DD, Sheehan JP; Diabetes Multicenter Research Group. Efficacy, safety, and tolerability of once-daily niacin for the treatment of dyslipidemia associated with type 2 diabetes: results of the assessment of diabetes control and evaluation of the efficacy of niaspan trial. Arch Intern Med. 2002 Jul 22;162(14):1568-76. PubMed PMID: 12123399.	competing risk / special population
203	Pan J, Lin M, Kesala RL, Van J, Charles MA. Niacin treatment of the atherogenic lipid profile and Lp(a) in diabetes. Diabetes Obes Metab. 2002 Jul;4(4):255-61.	competing risk / special population
204	Wink J, Giacoppe G, King J. Effect of very-low-dose niacin on high-density lipoprotein in patients undergoing long-term statin therapy. Am Heart J. 2002 Mar;143(3):514-8.	No CVD outcomes and follow up shorter than 6 months

© 2019 D’Andrea E et al. *JAMA Network Open.*

n.	References	Reason(s) for exclusion from qualitative and quantitative analysis
205	Sakai T, Kamanna VS, Kashyap ML. Niacin, but not gemfibrozil, selectively increases LP-AI, a cardioprotective subfraction of HDL, in patients with low HDL cholesterol. Arterioscler Thromb Vasc Biol. 2001 Nov;21(11):1783-9.	No CT - no original data
206	McKenney JM, McCormick LS, Schaefer EJ, Black DM, Watkins ML. Effect of niacin and atorvastatin on lipoprotein subclasses in patients with atherogenic dyslipidemia. Am J Cardiol. 2001 Aug 1;88(3):270-4.	No CVD outcomes and follow up shorter than 6 months
207	Guyton JR, Blazing MA, Hagar J, Kashyap ML, Knopp RH, McKenney JM, Nash DT, Nash SD. Extended-release niacin vs gemfibrozil for the treatment of low levels of high-density lipoprotein cholesterol. Niaspan-Gemfibrozil Study Group. Arch Intern Med. 2000 Apr 24;160(8):1177-84. PubMed PMID: 10789612.	No CVD outcomes and follow up shorter than 6 months
208	Zema MJ. Gemfibrozil, nicotinic acid and combination therapy in patients with isolated hypoalphalipoproteinemia: a randomized, open-label, crossover study. J Am Coll Cardiol. 2000 Mar 1;35(3):640-6.	No CVD outcomes and follow up shorter than 6 months
209	Spósito AC, Caramelli B, Serrano CV Jr, Mansur AP, Ramires JA. Effect of niacin and etofibrate association on subjects with coronary artery disease and serum high-density lipoprotein cholesterol <35 mg/dl. Am J Cardiol. 1999 Jan 1;83(1):98-100, A8.	No CVD outcomes and follow up shorter than 6 months
210	Goldberg AC. Clinical trial experience with extended-release niacin (Niaspan): dose-escalation study. Am J Cardiol. 1998 Dec 17;82(12A):35U-38U; discussion 39U-41U.	No CT - no original data
211	Knopp RH, Alagona P, Davidson M, Goldberg AC, Kafonek SD, Kashyap M, Sprecher D, Superko HR, Jenkins S, Marcovina S. Equivalent efficacy of a time-release form of niacin (Niaspan) given once-a-night versus plain niacin in the management of hyperlipidemia. Metabolism. 1998 Sep;47(9):1097-104.	No CVD outcomes and follow up shorter than 6 months
213	McKenney JM, McCormick LS, Weiss S, Koren M, Kafonek S, Black DM. A randomized trial of the effects of atorvastatin and niacin in patients with combined hyperlipidemia or isolated hypertriglyceridemia. Collaborative Atorvastatin Study Group. Am J Med. 1998 Feb;104(2):137-43.	No CVD outcomes and follow up shorter than 6 months
214	Gardner SF, Marx MA, White LM, Granberry MC, Skelton DR, Fonseca VA. Combination of low-dose niacin and pravastatin improves the lipid profile in diabetic patients without compromising glycemic control. Ann Pharmacother. 1997 Jun;31(6):677-82. PubMed PMID: 9184704.	competing risk / special population
215	Mostaza JM, Schulz I, Vega GL, Grundy SM. Comparison of pravastatin with crystalline nicotinic acid monotherapy in treatment of combined hyperlipidemia. Am J Cardiol. 1997 May 1;79(9):1298-301. PubMed PMID: 9164913.	No CVD outcomes and follow up shorter than 6 months
216	Aronov DM, Keenan JM, Akhmedzhanov NM, Perova NV, Oganov RY, Kiseleva NY. Clinical trial of wax-matrix sustained-release niacin in a Russian population with hypercholesterolemia. Arch Fam Med. 1996 Nov-Dec;5(10):567-75. PubMed PMID: 8930228.	No CVD outcomes and follow up shorter than 6 months
217	Gardner SF, Schneider EF, Granberry MC, Carter IR. Combination therapy with low-dose lovastatin and niacin is as effective as higher-dose lovastatin. Pharmacotherapy. 1996 May-Jun;16(3):419-23. PubMed PMID: 8726600.	No CVD outcomes and follow up shorter than 6 months
218	O'Keefe JH Jr, Harris WS, Nelson J, Windsor SL. Effects of pravastatin with niacin or magnesium on lipid levels and postprandial lipemia. Am J Cardiol. 1995 Sep 1;76(7):480-4. PubMed PMID: 7653448.	No CVD outcomes and follow up shorter than 6 months
219	Lal SM, Hewett JE, Petroski GF, Van Stone JC, Ross G Jr. Effects of nicotinic acid and lovastatin in renal transplant patients: a prospective, randomized, open-labeled crossover trial. Am J Kidney Dis. 1995 Apr;25(4):616-22.	competing risk / special population
220	Vacek JL, Dittmeier G, Chiarelli T, White J, Bell HH. Comparison of lovastatin (20 mg) and nicotinic acid (1.2 g) with either drug alone for type II hyperlipoproteinemia. Am J Cardiol. 1995 Jul 15;76(3):182-4.	No CVD outcomes and follow up shorter than 6 months
221	Avogaro P, Bittolo-Bon G, Pais M, Taroni GC. Effect of a new niacin derivative (nicotinic hexaester of D-glucitol) on type IIA, IIB and IV hyperlipoproteinemia in man. Pharmacol Res Commun. 1977 Jun;9(6):599-606.	No outcome of interest
222	King JM, Crouse JR, Terry JG, Morgan TM, Spray BJ, Miller NE. Evaluation of effects of unmodified niacin on fasting and postprandial plasma lipids in normolipidemic men with hypoalphalipoproteinemia. Am J Med. 1994 Oct;97(4):323-31. PubMed PMID: 7942933.	No CVD outcomes and follow up shorter than 6 months

© 2019 D’Andrea E et al. *JAMA Network Open.*

n.	References	Reason(s) for exclusion from qualitative and quantitative analysis
223	Tsalamandris C, Panagiotopoulos S, Sinha A, Cooper ME, Jerums G. Complementary effects of pravastatin and nicotinic acid in the treatment of combined hyperlipidaemia in diabetic and non-diabetic patients. J Cardiovasc Risk. 1994 Oct;1(3):231-9.	No CVD outcomes and follow up shorter than 6 months
224	Illingworth DR, Stein EA, Mitchel YB, Dujovne CA, Frost PH, Knopp RH, Tun P, Zupkis RV, Greguski RA. Comparative effects of lovastatin and niacin in primary hypercholesterolemia. A prospective trial. Arch Intern Med. 1994 Jul 25;154(14):1586-95.	No CVD outcomes and follow up shorter than 6 months
225	Jacobson TA, Chin MM, Fromell GJ, Jokubaitis LA, Amorosa LF. Fluvastatin with and without niacin for hypercholesterolemia. Am J Cardiol. 1994 Jul 15;74(2):149-54. PubMed PMID: 8023779.	No CVD outcomes and follow up shorter than 6 months
226	McKenney JM, Proctor JD, Harris S, Chinchili VM. A comparison of the efficacy and toxic effects of sustained- vs immediate-release niacin in hypercholesterolemic patients. JAMA. 1994 Mar 2;271(9):672-7. PubMed PMID: 8309029.	No comparison of interest
227	Seed M, O'Connor B, Perombelon N, O'Donnell M, Reaveley D, Knight BL. The effect of nicotinic acid and acipimox on lipoprotein(a) concentration and turnover. Atherosclerosis. 1993 Jun;101(1):61-8. PubMed PMID: 8216503.	No CT - no original data
228	O'Kane MJ, Trinick TR, Tynan MB, Trimble ER, Nicholls DP. A comparison of acipimox and nicotinic acid in type 2b hyperlipidaemia. Br J Clin Pharmacol. 1992 Apr;33(4):451-3. PubMed PMID: 1576071; PubMed Central PMCID: PMC1381338.	No CVD outcomes and follow up shorter than 6 months
229	Keenan JM, Wenz JB, Ripsin CM, Huang Z, McCaffrey DJ. A clinical trial of oat bran and niacin in the treatment of hyperlipidemia. J Fam Pract. 1992 Mar;34(3):313-9. PubMed PMID: 1541958.	No CVD outcomes and follow up shorter than 6 months
230	Keenan JM, Fontaine PL, Wenz JB, Myers S, Huang ZQ, Ripsin CM. Niacin revisited. A randomized, controlled trial of wax-matrix sustained-release niacin in hypercholesterolemia. Arch Intern Med. 1991 Jul;151(7):1424-32.	No CVD outcomes and follow up shorter than 6 months
231	Canner PL, Berge KG, Wenger NK, Stamler J, Friedman L, Prineas RJ, Friedewald W. Fifteen year mortality in Coronary Drug Project patients: long-term benefit with niacin. J Am Coll Cardiol. 1986 Dec;8(6):1245-55.	Follow-up study
232	Knopp RH, Ginsberg J, Albers JJ, Hoff C, Ogilvie JT, Warnick GR, Burrows E, Retzlaff B, Poole M. Contrasting effects of unmodified and time-release forms of niacin on lipoproteins in hyperlipidemic subjects: clues to mechanism of action of niacin. Metabolism. 1985 Jul;34(7):642-50. PubMed PMID: 3925290.	No comparison of interest
233	Hoeg JM, Maher MB, Bou E, Zech LA, Bailey KR, Gregg RE, Sprecher DL, Susser JK, Pikus AM, Brewer HB Jr. Normalization of plasma lipoprotein concentrations in patients with type II hyperlipoproteinemia by combined use of neomycin and niacin. Circulation. 1984 Dec;70(6):1004-11. PubMed PMID: 6388897.	No CT - no original data
234	Paoletti R, Franceschini G, Sirtori CR. Influence of bezafibrate, fenofibrate, nicotinic acid and etofibrate on plasma high-density lipoprotein levels. Am J Cardiol. 1983 Aug 22;52(4):21B-27B.	No CT - no original data
235	Kane JP, Malloy MJ, Tun P, Phillips NR, Freedman DD, Williams ML, Rowe JS, Havel RJ. Normalization of low-density-lipoprotein levels in heterozygous familial hypercholesterolemia with a combined drug regimen. N Engl J Med. 1981 Jan 29;304(5):251-8. PubMed PMID: 7003391.	No niacin - combined regimen
236	Karas RH, Kashyap ML, Knopp RH, Keller LH, Bajorunas DR, Davidson MH. Long-term safety and efficacy of a combination of niacin extended release and simvastatin in patients with dyslipidemia: the OCEANS study. Am J Cardiovasc Drugs. 2008;8(2):69-81. ClinicalTrials.gov Identifier: NCT00080275	competing risk / special population
237	CPC study: Zhao XQ, Phan BA, Chu B, Bray F, Moore AB, Polissar NL, Dodge JT, Jr., Lee CD, Hatsukami TS, Yuan C. Testing the hypothesis of atherosclerotic plaque lipid depletion during lipid therapy by magnetic resonance imaging: Study design of carotid plaque composition study. Am Heart J. 2007;154:239-246	No CVD outcomes
238	Cheung MC, Zhao XQ, Chait A, Albers JJ, Brown BG. Antioxidant supplements block the response of HDL to simvastatin-niacin therapy in patients with coronary artery disease and low HDL. Arterioscler Thromb Vasc Biol. 2001 Aug;21(8):1320-6.	No outcome of interest

© 2019 D’Andrea E et al. *JAMA Network Open.*

n.	References	Reason(s) for exclusion from qualitative and quantitative analysis
240	Kent S, Haynes R, Hopewell JC, Parish S, Gray A, Landray MJ, Collins R, Armitage J, Mihaylova B; HPS2-THRIVE Collaborative Group. Effects of Vascular and Nonvascular Adverse Events and of Extended-Release Niacin With Laropiprant on Health and Healthcare Costs. Circ Cardiovasc Qual Outcomes. 2016 Jul;9(4):348-54. ClinicalTrials.gov Identifier: NCT00461630	No CT - no original data
241	Barter PJ, Nicholls S, Rye KA, Anantharamaiah GM, Navab M, Fogelman AM. Antiinflammatory properties of HDL. Circ Res. 2004 Oct 15;95(8):764-72. Review. ClinicalTrials.gov identifier NCT00508989	No CT - no original data
242	Taylor AJ, Villines TC, Stanek EJ. Paradoxical progression of atherosclerosis related to low-density lipoprotein reduction and exposure to ezetimibe. Eur Heart J. 2012 Dec;33(23):2939-45. doi: 10.1093/eurheartj/ehs105. Epub 2012 May 7. ClinicalTrials.gov Identifier: NCT00397657	No niacin
243	Wooten JS, Nambi P, Gillard BK, Pownall HJ, Coraza I, Scott LW, Nambi V, Ballantyne CM, Balasubramanyam A. Intensive lifestyle modification reduces Lp-PLA2 in dyslipidemic HIV/HAART patients. Med Sci Sports Exerc. 2013 Jun;45(6):1043-50. ClinicalTrials.gov NCT00246376.	competing risk / special population
244	Brown BG, Cheung MC, Lee AC, Zhao XQ, Chait A. Antioxidant vitamins and lipid therapy: end of a long romance? Arterioscler Thromb Vasc Biol. 2002 Oct 1;22(10):1535-46. Review.	No CT - no original data
245	Vu CN, Ruiz-Esponda R, Yang E, Chang E, Gillard B, Pownall HJ, Hoogeveen RC, Coraza I, Balasubramanyam A. Altered relationship of plasma triglycerides to HDL cholesterol in patients with HIV/HAART-associated dyslipidemia: further evidence for a unique form of metabolic syndrome in HIV patients. Metabolism. 2013 Jul;62(7):1014-20. ClinicalTrials.gov NCT00246376	competing risk / special population
246	Kwiterovich PO Jr. The antiatherogenic role of high-density lipoprotein cholesterol. Am J Cardiol. 1998 Nov 5;82(9A):13Q-21Q. Review. ClinicalTrials.gov identifier NCT00508989	No CT - no original data
247	Viswambharan H, Ming XF, Zhu S, Hubsch A, Lerch P, Vergères G, Rusconi S, Yang Z. Reconstituted high-density lipoprotein inhibits thrombin-induced endothelial tissue factor expression through inhibition of RhoA and stimulation of phosphatidylinositol 3-kinase but not Akt/endothelial nitric oxide synthase. Circ Res. 2004 Apr 16;94(7):918-25. Epub 2004 Feb 26. ClinicalTrials.gov identifier NCT00508989	No niacin
248	Guyton JR, Fazio S, Adewale AJ, Jensen E, Tomassini JE, Shah A, Tershakovec AM. Effect of extended-release niacin on new-onset diabetes among hyperlipidemic patients treated with ezetimibe/simvastatin in a randomized controlled trial. Diabetes Care. 2012 Apr;35(4):857-60. doi: 10.2337/dc11-1369. Epub 2012 Feb 14. Clinicaltrials.gov Identifier: NCT00271817	No CT - no original data
249	Guyton JR, Goldberg AC, Kreisberg RA, Sprecher DL, Superko HR, O'Connor CM. Effectiveness of once-nightly dosing of extended-release niacin alone and in combination for hypercholesterolemia. Am J Cardiol. 1998 Sep 15;82(6):737-43. ClinicalTrials.gov Identifier: NCT00127218	No CT - no original data
250	Ballantyne CM, Herd JA, Ferlic LL, Dunn JK, Farmer JA, Jones PH, Schein JR, Gotto AM Jr. Influence of low HDL on progression of coronary artery disease and response to fluvastatin therapy. Circulation. 1999 Feb 16;99(6):736-43. ClinicalTrials.gov Identifier: NCT00127218	No CT - no original data
251	Brown BG, Zhao XQ, Chait A, Fisher LD, Cheung MC, Morse JS, Dowdy AA, Marino EK, Bolson EL, Alaupovic P, Frohlich J, Albers JJ. Simvastatin and niacin, antioxidant vitamins, or the combination for the prevention of coronary disease. N Engl J Med. 2001 Nov 29;345(22):1583-92.	No CT - no original data
252	Cheung MC, Wolfbauer G, Brown BG, Albers JJ. Relationship between plasma phospholipid transfer protein activity and HDL subclasses among patients with low HDL and cardiovascular disease. Atherosclerosis. 1999 Jan;142(1):201-5. ClinicalTrials.gov NCT00000553.	No niacin
253	Brown BG, Zhao XQ, Chait A, Frohlich J, Cheung M, Heise N, Dowdy A, DeAngelis D, Fisher LD, Albers J. Lipid altering or antioxidant vitamins for patients with coronary disease and very low HDL cholesterol? The HDL-Atherosclerosis Treatment Study Design. Can J Cardiol. 1998 Apr;14 Suppl A:6A-13A. Review. ClinicalTrials.gov NCT00000553.	No CT - no original data
254	Brown G, Stewart BF, Zhao XQ, Hillger LA, Poulin D, Albers JJ. What benefit can be derived from treating normocholesterolemic patients with coronary artery disease? Am J Cardiol. 1995 Sep 28;76(9):93C-97C. Review. ClinicalTrials.gov NCT00000553.	No CT - no original data
255	Morgan, J.M, Capuzzi, D.M, Guyton, J.R, Centor, R.M, Goldberg, R, Robbins, D.C, DiPette, D, Jenkins, S, and Marcovina, S. Treatment effect of Niaspan, a controlled-release niacin, in patients with hypercholesterolemia (a placebo-controlled trial) . J Cardiovasc Pharmacol Therapeut. 1996; 1: 195–202	No CVD outcomes and follow up shorter than 6 months

© 2019 D’Andrea E et al. *JAMA Network Open.*

n.	References	Reason(s) for exclusion from qualitative and quantitative analysis
256	Capuzzi DM, Morgan JM, Weiss RJ, Chitra RR, Hutchinson HG, Cressman MD. Beneficial effects of rosuvastatin alone and in combination with extended-release niacin in patients with a combined hyperlipidemia and low high-density lipoprotein cholesterol levels. Am J Cardiol. 2003 Jun 1;91(11):1304-10. Trial Number: 4522IL/0029	No CVD outcomes
257	Lauring B , Taggart AK , Tata JR , Dunbar R , Caro L , Cheng K , Chin J , Colletti SL , Cote J , Khalilieh S , Liu J , Luo WL , Maclean AA , Peterson LB , Polis AB , Sirah W , Wu TJ , Liu X , Jin L , Wu K , Boatman PD , Semple G , Behan DP , Connolly DT , Lai E , Wagner JA , Wright SD , Cuffie C , Mitchel YB , Rader DJ , Paolini JF , Waters MG and Plump A Niacin lipid efficacy is independent of both the niacin receptor GPR109A and free fatty acid suppression. Science translational medicine, 2012, 4(148), 148ra115	No outcome of interest
258	Usman MH , Qamar A , Gadi R , Lilly S , Goel H , Hampson J , Mucksavage ML , Nathanson GA , Rader DJ and Dunbar RL. Extended-release niacin acutely suppresses postprandial triglyceridemia. The American journal of medicine, 2012, 125(10), 1026	No outcome of interest
259	Sazonov V , Maccubbin D , Sisk CM and Canner PL. Effects of niacin on the incidence of new onset diabetes and cardiovascular events in patients with normoglycaemia and impaired fasting glucose. International journal of clinical practice, 2013, 67(4), 297	No CT - no original data
260	Paolini JF , Mitchel YB , Reyes R , Thompson-Bell S , Yu Q , Lai E , Watson DJ , Norquist JM , Sisk CM and Bays HE. Measuring flushing symptoms with extended-release niacin using the flushing symptom questionnaire: results from a randomised placebo-controlled clinical trial. International journal of clinical practice, 2008, 62(6), 896	No outcome of interest
261	Gleim G , Liu N , Thompson-Bell S , McCrary Sisk C , Pasternak RC , Mitchel Y , Paolini JF and Ballantyne CM. Efficacy and safety profile of co-administered ER niacin /laropiprant and simvastatin in dyslipidaemia. British journal of cardiology, 2009, 16(2), 90. ClinicalTrials.gov Identifier: NCT00269217	No CVD outcomes and follow up shorter than 6 months
262	Karacaglar E , Atar I , Altin C , Yetis B , Cakmak A , Bayraktar N , Ozin B and Muderrisoglu H. The effects of niacin on HsCRP in patients with non ST elevated acute coronary syndrome. International journal of cardiology, 2010, 140, S25	competing risk / special population
263	Thachil R , Carson PS and Banka SS. Niaspirin: Combinations of niacin with aspirin to reduce niacin induced flush. Cardiology (Switzerland), 2014, 128, 266	No outcome of interest
264	Karacaglar E , Atar I , Altin C , Yetis B , Cakmak A , Bayraktar N , Coner A , Ozin B and Muderrisoglu H. The effects of niacin on inflammation in patients with non-ST elevated acute coronary syndrome. Acta Cardiologica Sinica, 2015, 31(2), 120	No CVD outcomes and follow up shorter than 6 months
265	Asleh R , Farbstein D , Blum S , Vardi M , Shalom H , Saraf S , O'Brien K , Marcovina S , Roberts BH , Herrington D , Robinson JG and Levy AP. HDL-induced cholesterol efflux capacity from niacin therapy in diabetes is haptoglobin genotype-dependent. Circulation, 2014, 130	competing risk / special population
266	Rao M , Steffes M , Bostom A and Ix JH. Effect of niacin on FGF23 concentration in chronic kidney disease. American journal of nephrology, 2014, 39(6), 484	competing risk / special population
267	McBride R. The role of niacin in raising high-density lipoprotein cholesterol to reduce cardiovascular events in patients with atherosclerotic cardiovascular disease and optimally treated low-density lipoprotein cholesterol: Rationale and study design. the Atherothrombosis Intervention in Metabolic syndrome with low HDL/high triglycerides: Impact on Global Health outcomes (AIM-HIGH). American heart journal, 2011, 161(3), 471	No CT - no original data
268	Elbaz M , Lhermusier T , Bataille V , Bongard V , Roncalli J , Bura-Riviere A , Ferrieres J , Carrie D , Galinier M and Cambou J-P. Impact of Niacin therapy on endothelial vasomotricity in patients with low HDL shortly after an acute coronary syndrome. Archives of Cardiovascular Diseases Supplements, 2011, 3(1), 10	No CVD outcomes and follow up shorter than 6 months
269	Lin C , Grandinetti A , Shikuma C , Souza S , Parikh N , Nakamoto B , Kallianpur KJ and Chow D The effects of extended release niacin on lipoprotein sub-particle concentrations in HIV-infected patients. Hawai'i journal of medicine & public health : a journal of Asia Pacific Medicine & Public Health, 2013, 72(4), 123	competing risk / special population
270	Thakkar RB , Kashyap ML , Lewin AJ , Krause SL , Jiang P and Padley RJ. Acetylsalicylic acid reduces niacin extended-release-induced flushing in patients with dyslipidemia. Am j card drugs, 2009, 9(2), 69	No outcome of interest

© 2019 D’Andrea E et al. *JAMA Network Open.*

n.	References	Reason(s) for exclusion from qualitative and quantitative analysis
271	Daniel Keene D , Price C , Shun-Shin M and Francis D. The observational HDL hypothesis: A useful therapeutic target? A meta-analysis of 117,411 patients in randomised controlled trials. European Journal of Preventive Cardiology, 2014, 21(1 SUPPL. 1), S121	No CT
272	Asghar J , Gill KU , Murad S , Asif S , Bashir A and Mahmood G. Biostatistical analysis of morbidity and Mortality due to myocardial infarction and its prevention by hypolipidemic drug regimen. Pakistan Journal of Medical and Health Sciences, 2011, 5(3), 445	No CT - no original data
273	Ferchaud-Roucher V , Croyal M , Krempf M and Ouguerram K. Plasma lipidome characterization using UHPLC-HRMS and ion mobility of hypertriglyceridemic patients on nicotinic acid. Atherosclerosis, 2015, 241(1), e123	No CVD outcomes and follow up shorter than 6 months
274	Benefit of Elevation of HDL-C on Cardiovascular Outcomes in Women. ClinicalTrials.gov Identifier: NCT00590629	No CVD outcomes and follow up shorter than 6 months
275	Influence of Combined Therapy of Niacin and Statins on Stem Cell Mobilization and Inflammatory Parameters in Patients Suffering From Coronary Artery Disease. Niacin As Secondary Prevention Of Coronary Artery Disease (NASPOCAD). ClinicalTrials.gov Identifier: NCT00431145	No results
276	A Study of AMR101 to Evaluate Its Ability to Reduce Cardiovascular Events in High Risk Patients With Hypertriglyceridemia and on Statin. The Primary Objective is to Evaluate the Effect of 4 g/Day AMR101 for Preventing the Occurrence of a First Major Cardiovascular Event. ClinicalTrials.gov Identifier: NCT01492361	No niacin
277	The Low HDL On Six Weeks Statin Therapy (LOW) Study. ClinicalTrials.gov Identifier: NCT00238004	No CVD outcomes and follow up shorter than 6 months
278	Retrospective Study of the Effectiveness and Safety of Niacin and Steroid Eye Drops for Retinal Vein Occlusions.	No outcome of interest
279	The Dose Response of Niacin ER/Lovastatin on Peak Walking Time (PWT) in Patients With Intermittent Claudication – TROPIC. ClinicalTrials.gov Identifier: NCT00071266	No results
281	Insull Jr W , Basile JN , Vo AN , Jiang P , Thakkar R and Padley RJ. Efficacy and safety of combination therapy with niacin extended-release and simvastatin versus atorvastatin in patients with dyslipidemia: The SUPREME Study. Journal of clinical lipidology, 2009, 3(2), 109	No CVD outcomes and follow up shorter than 6 months
282	Devasia T , Rao Y , Shenoy G , Razzak A and Rajaram P. Efficacy and safety of a combination therapy of statin and krill oil versus statin and niacin in dyslipidemia: A randomized, open, comparator study. Indian heart journal, 2013, 65, S85. http://www.ijbcp.com/index.php/ijbcp/article/viewFile/977/881.	No CVD outcomes and follow up shorter than 6 months
283	Merck Carotid Atherosclerosis Trial (MK-0000-111)(COMPLETED) (MCAT). ClinicalTrials.gov Identifier: NCT00804843	No CVD outcomes and follow up shorter than 6 months
284	Comparative Efficacy Evaluation of Lipids When Treated With Niaspan & Statin or Other Lipid-Modifying Therapies-COMPELL. NCT00079638	No CT - no original data
285	Evaluation of the Effect of NICOtinic Acid (Niacin) on Elevated Lipoprotein(a) Levels (NICOLa Study). NCT00633698	No results
316	Bregar U, Jug B, Keber I, Cevc M, Sebestjen M. Extended-release niacin/laropiprant improves endothelial function in patients after myocardial infarction. Heart Vessels. 2014 May;29(3):313-9.	No CVD outcomes and follow up shorter than 6 months
354	Bays HE, McGovern ME. Once-daily niacin extended release/lovastatin combination suppsut has more favorable effects on lipoprotein particle size and subclass distribution than atorvastatin and simvastatin. Prev Cardiol. 2003;6:179- 88. [PMID: 14605511]	No CVD outcomes and follow up shorter than 6 months
363	Stein EA, Davidson MH, Dujovne CA, Hunninghake DB, Goldberg RB, Illingworth DR, et al. Efficacy and tolerability of low-dose simvastatin and niacin, alone and in combination, in patients with combined hyperlipidemia: a prospective trial. J Cardiovasc Pharmacol Ther. 1996;1:107-16. [PMID: 10684407]	No comparison of interest
373	Ballantyne CM, Davidson MH, McKenney J. Comparison of the safety and efficacy of a combination tablet of niacin extended release and simvastatin with simvastatin 80 mg monotherapy: the SEACOAST II (high-dose) study. J Clin Lipidol. 2008;2:79-90	No CT - no original data

© 2019 D’Andrea E et al. *JAMA Network Open.*

n.	References	Reason(s) for exclusion from qualitative and quantitative analysis
375	Jafri H, Alsheikh-Ali AA, Mooney P, Kimmelstiel CD, Karas RH, Kuvin JT. Extended-release niacin reduces LDL particle number without changing total LDL cholesterol in patients with stable CAD. J Clin Lipidol. 2009;3:45–50.	No CVD outcomes and follow up shorter than 6 months
379	Evaluation of the safety and efficacy of Advicor (a combination tablet of niacin extended-release/lovastatin immediate-release): a dose response study. FDA-Advicor (MA-14). Silver Spring, MD: U.S. Food and Drug Administra-tion; 2008. (handsearch)	No CT
380	Evaluation of the safety and efficacy of Advicor (a combination tablet of niacin extended-release/lovastatin immediate-release): a dose ranging study. FDA- Advicor (M-06). Silver Spring, MD: U.S. Food and Drug Administration; 2008. (handsearch)	No outcome of interest
381	Maccubbin D, Bays HE, Olsson AG, Elinoff V, Elis A, Mitchel Y, et al. Lipid-modifying efficacy and tolerability of extended-release niacin/laropiprant in patients with primary hypercholesterolaemia or mixed dyslipidaemia. International Journal of Clinical Practice 2008;62:1959–70. ClinicalTrials.gov: NCT00269204	No CVD outcomes and follow up shorter than 6 months
382	Goldberg A, Alagona P Jr, Capuzzi DM, Guyton J, Morgan JM, Rodgers J, Sachson R, Samuel P. Multiple-dose efficacy and safety of an extended-release form of niacin in the management of hyperlipidemia. Am J Cardiol. 2000 May 1;85(9):1100-5. PubMed PMID: 10781759.	No CVD outcomes
383	Hunninghake DB, McGovern ME, Koren M, Brazg R, Murdock D, Weiss S, Pearson T. A dose-ranging study of a new, once-daily, dual-component drug product containing niacin extended-release and lovastatin. Clin Cardiol. 2003 Mar;26(3):112-8. PubMed PMID: 12685616.	No CVD outcomes
384	Thoenes M, Oguchi A, Nagamia S, Vaccari CS, Hammoud R, Umpierrez GE, Khan BV. The effects of extended-release niacin on carotid intimal media thickness, endothelial function and inflammatory markers in patients with the metabolic syndrome. Int J Clin Pract. 2007 Nov;61(11):1942-8.	No CVD outcomes
385	Santanu G, Suhrita P, Mookerjee S, Tania K, Mita S, Pramit G, Sharmila G, Miraj M, Debdutta M. Lipid modifying action of atorvastatin in comparison to combination of atorvastatin and nicotinic acid in patients with ischaemic heart disease. Indian Heart J. 2011 Sep-Oct;63(5):434-7.	No CVD outcomes
386	Ballantyne CM, Davidson MH, McKenney J, Keller LH, Bajorunas DR, Karas RH. Comparison of the safety and efficacy of a combination tablet of niacin extended release and simvastatin vs simvastatin monotherapy in patients with increased non-HDL cholesterol (from the SEACOAST I study). Am J Cardiol. 2008 May 15;101(10):1428-36. ClinicalTrials.gov Identifier: NCT00082251	No CVD outcomes
387	Airan-Javia SL, Wolf RL, Wolfe ML, Tadesse M, Mohler E, Reilly MP. Atheroprotective lipoprotein effects of a niacin-simvastatin combination compared to low- and high-dose simvastatin monotherapy. Am Heart J. 2009 Apr;157(4):687.e1-8.	No CVD outcomes
388	Capuzzi DM, Morgan JM, Carey CM, Intenzo C, Tulenko T, Kearney D, Walker K, Cressman MD. Rosuvastatin alone or with extended-release niacin: a new therapeutic option for patients with combined hyperlipidemia. Prev Cardiol. 2004 Fall;7(4):176-81. Trial Number: 4522IL/0029	No CT - no original data
389	Nessim SA, Chin HP, Alaupovic P, Blankenhorn DH. Combined therapy of niacin, colestipol, and fat-controlled diet in men with coronary bypass. Effect on blood lipids and apolipoproteins. Arteriosclerosis. 1983 Nov-Dec;3(6):568-73.	No CVD outcomes
390	Lee JM, Robson MD, Yu LM, Shirodaria CC, Cunnington C, Kylintireas I, Digby JE, Bannister T, Handa A, Wiesmann F, Durrington PN, Channon KM, Neubauer S, Choudhury RP. Effects of high-dose modified-release nicotinic acid on atherosclerosis and vascular function: a randomized, placebo-controlled, magnetic resonance imaging study. J Am Coll Cardiol. 2009 Nov 3;54(19):1787-94. Oxford Niaspan Study: Effects of Niaspan on Atherosclerosis and Endothelial Function; NCT00232531	No CVD outcomes
391	Harikrishnan S, Rajeev E, Tharakan JA, Titus T, Ajit Kumar VK, Sivasankaran S, Krishnamoorthy KM, Nair K. Efficacy and safety of combination of extended release niacin and atorvastatin in patients with low levels of high density lipoprotein cholesterol. Indian Heart J. 2008 May-Jun;60(3):215-22.	No CVD outcomes
392	Kashyap ML, McGovern ME, Berra K, Guyton JR, Kwiterovich PO, Harper WL, Toth PD, Favrot LK, Kerzner B, Nash SD, Bays HE, Simmons PD. Long-term safety and efficacy of a once-daily niacin/lovastatin formulation for patients with dyslipidemia. Am J Cardiol. 2002 Mar 15;89(6):672-8. PubMed PMID: 11897208.	No CVD outcomes

© 2019 D’Andrea E et al. *JAMA Network Open.*

n.	References	Reason(s) for exclusion from qualitative and quantitative analysis
393	162. Elam MB, Hunninghake DB, Davis KB, Garg R, Johnson C, Egan D, Kostis JB, Sheps DS, Brinton EA. Effect of niacin on lipid and lipoprotein levels and glycemic control in patients with diabetes and peripheral arterial disease: the ADMIT study: A randomized trial. Arterial Disease Multiple Intervention Trial. JAMA. 2000 Sep 13;284(10):1263-70. ADMIT study – included only data on patients without diabetes – excluded measurement at 18-week	competing risk / special population
412	Brown BG, Bardsley J, Poulin D, et al. Moderate dose, three-drug therapy with niacin, lovastatin, and colestipol to reduce low-density lipoprotein cholesterol 100 mg/dl in patients with hyperlipidemia and coronary artery disease. Am J Cardiol 1997;80:111–5.	No CVD outcomes
413	Vega GL, Grundy SM. Lipoprotein responses to treatment with lovastatin, gemfibrozil, and nicotinic acid in normolipidemic patients with hypoalphalipoproteinemia. Arch Intern Med 1994;154:73– 82.	No CVD outcomes and follow up shorter than 6 months
414	Lavie CJ, Mailander L, Milani RV. Marked benefit with sustained- release niacin therapy in patients with "isolated" very low levels of high-density lipoprotein cholesterol and coronary artery disease. Am J Cardiol 1992;69:1083–5.	No CVD outcomes and follow up shorter than 6 months
415	Kuvin JT, Ramet ME, Patel AR, Pandian NG, Mendelsohn ME, Karas RH. A novel mechanism for the beneficial vascular effects of high-density lipoprotein cholesterol: enhanced vasorelaxation and increased endothelial nitric oxide synthase expression. Am Heart J 2002;144:165–72.	No CVD outcomes and follow up shorter than 6 months
416	Ball MJ, Vella M, Rechlass JP, et al. Acipimox in the treatment of patients with hyperlipidaemia: a double blind trial. Eur J Clin Pharmacol 1986;31:201– 4.	No CVD outcomes and follow up shorter than 6 months
417	Crepaldi G, Avogaro P, Descovich GC, et al. Plasma lipid lowering activity of acipimox in patients with type II and type IV hyperlipo- proteinemia. Results of a multicenter trial. Atherosclerosis 1988;70: 115–21.	No CVD outcomes and follow up shorter than 6 months
418	Davoren PM, Kelly W, Gries FA, Hubinger A, Whately-Smith C, Alberti KG. Long-term effects of a sustained-release preparation of acipimox on dyslipidemia and glucose metabolism in non-insulin- dependent diabetes mellitus. Metabolism 1998;47:250–6.	competing risk / special population
419	Dean JD, McCarthy S, Betteridge DJ, Whately-Smith C, Powell J, Owens DR. The effect of acipimox in patients with type 2 diabetes and persistent hyperlipidaemia. Diabetes Med 1992;9:611–5.	competing risk / special population
420	Fulcher GR, Catalano C, Walker M, et al. A double blind study of the effect of acipimox on serum lipids, blood glucose control and insulin action in non-obese patients with type 2 diabetes mellitus. Diabetes Med 1992;9:908–14.	competing risk / special population
421	Koev D, Zlateva S, Susic M, et al. Improvement of lipoprotein lipid composition in type II diabetic patients with concomitant hyperlipo- proteinemia by acipimox treatment. Results of a multicenter trial. Diabetes Care 1993;16:1285–90.	competing risk / special population
422	Otto C, Parhofer KG, Ritter MM, Richter WO, Schwandt P. Effects of acipimox on haemorheology and plasma lipoproteins in patients with mixed hyperlipoproteinaemia. Br J Clin Pharmacol 1998;46: 473– 8.	No CVD outcomes and follow up shorter than 6 months
423	Taskinen MR, Nikkila EA. Effects of acipimox on serum lipids, lipoproteins and lipolytic enzymes in hypertriglyceridemia. Athero- sclerosis 1988;69:249–55.	No CVD outcomes
424	Vaag AA, Beck-Nielsen H. Effects of prolonged Acipimox treatment on glucose and lipid metabolism and on in vivo insulin sensitivity in patients with non-insulin dependent diabetes mellitus. Acta Endo- crinol (Copenh) 1992;127:344–50.	competing risk / special population
425	Carotid IMT (Intima Media Thickening) Study (0524A-041) (TERMINATED) (ACHIEVE). Merck Sharp & Dohme Corp. 2009. ClinicalTrials.gov (NCT00384293)	No results
426	Lee K, Ahn TH, Kang WC, Han SH, Choi IS, Shin EK. The effects of statin and niacin on plaque stability, plaque regression, inflammation and oxidative stress in patients with mild to moderate coronary artery stenosis. Korean Circulation Journal 2011;41:641–8.	No CVD outcomes
427	Moore A, Phan BA, Challender C, et al. Effects of adding extended-release niacin and colesevelam to statin therapy on lipid levels in subjects with atherosclerotic disease. J Clin Lipidol 2007;1:620–5.	No CVD outcomes
428	A Multicenter, Randomized, Double-Blind, "Crossover" Design Study to Evaluate the Lipid-Altering Efficacy and Safety of MK-0524B Combination Tablet Compared to MK-0524A + Simvastatin Coadministration in Patients With Primary Hypercholesterolemia and Mixed Dyslipidemia. Merck Sharp & Dohme Corp. 2016. ClinicalTrials.gov (NCT00479882)	No CVD outcomes and follow up shorter than 6 months

© 2019 D'Andrea E et al. *JAMA Network Open.*

n.	References	Reason(s) for exclusion from qualitative and quantitative analysis
429	A Phase III Multicenter, Double-Blind, Crossover Design Study to Evaluate Lipid-Altering Efficacy and Safety of Extended-Release Niacin/Laropiprant/Simvastatin Combination Tablet in Patients With Primary Hypercholesterolemia or Mixed Dyslipidemia. Merck Sharp & Dohme Corp. 2016. ClinicalTrials.gov (NCT01294683)	No CVD outcomes and follow up shorter than 6 months
430	A Multicenter, Randomized, Double-Blind, Parallel Group, 12 Week Study to Evaluate the Efficacy and Safety of MK0524B Versus Atorvastatin in Patients With Mixed Hyperlipidemia. Merck Sharp & Dohme Corp. 2016. ClinicalTrials.gov (NCT00289900)	No CVD outcomes and follow up shorter than 6 months
CT, clinical trial; CVD, cardiovascular disease.		

© 2019 D’Andrea E et al. *JAMA Network Open.*

eTable 2. Definitions of cardiovascular outcomes evaluated in the meta-analyses and meta-regression analysis

Endpoints	Definitions	No. studies that documented outcome events [Trial name or First Author]
Cardiovascular disease (CVD) mortality	Overall deaths from coronary heart disease (*i.e.,* acute myocardial infarction, sudden death, or ischemic heart failure event), cerebrovascular events, or revascularization procedures.	17 [VA drug, CDP, CLAS I, STOCKHOLM, FATS, UCSF-SCOR, HARP, PAST, ARBITER–2, AFREGS, Guyton et al, SANG, ARBITER-6-HALTS, AIM-HIGH, NIA plaque, ELIMIT, HPS2-THRIVE]
Coronary heart disease mortality	Overall deaths from myocardial infarction, sudden death, or heart failure event.	9 [CDP, STOCKHOLM, FATS, UCSF-SCOR, HARP, AFREGS, Guyton et al, AIM-HIGH, HPS2-THRIVE]
Acute coronary syndrome	Fatal and non-fatal myocardial infarction, hospitalization for unstable angina.	16 [VA drug, CDP, CLAS I, STOCKHOLM, FATS, UCSF-SCOR, HARP, PAST, ARBITER–2, AFREGS, Guyton et al, SANG, ARBITER-6-HALTS, AIM-HIGH, ELIMIT, HPS2-THRIVE]
Stroke	Fatal and non-fatal stroke*	11 [CDP, STOCKHOLM, FATS, UCSF-SCOR, ARBITER – 2, AFREGS, Guyton et al, AIM-HIGH, NIA plaque, ELIMIT, HPS2-THRIVE]
Revascularization procedures	Coronary and non-coronary revascularization procedures.	13 [CDP, CLAS I, FATS, HARP, PAST, ARBITER – 2, AFREGS, SANG, ARBITER-6-HALTS, AIM-HIGH, NIA plaque, ELIMIT, HPS2-THRIVE]
Major Adverse events (MACE)	Overall deaths from coronary heart disease, nonfatal myocardial infarction, hospitalization for angina, stroke, revascularization procedures.	17 [VA drug, CDP, CLAS I, STOCKHOLM, FATS, UCSF-SCOR, HARP, PAST, ARBITER–2, AFREGS, Guyton et al, SANG, ARBITER-6-HALTS, AIM-HIGH, NIA plaque, ELIMIT, HPS2-THRIVE]

CVD, cardiovascular disease; MACE, major adverse cardiovascular events; VA drug, Veterans Affairs drug study; CDP, Coronary Drug Project; CLAS I, Cholesterol-Lowering Atherosclerosis Study I; STOCKHOLM, Stockholm Ischaemic Heart Disease Secondary prevention study; FATS, Familial Atherosclerosis Treatment Study; UCSF-SCOR, University of California San Francisco - Arteriosclerosis Specialized Center of Research Intervention Trial; HARP, Heart and Renal Protection study; PAST, Prevenzione Aterosclerosi Studio Torino; ARBITER-2, Arterial Biology for the Investigation of the Treatment Effects of Reducing Cholesterol trial; AFREGS, Armed Forces Regression Study; ARBITER 6-HALTS, Arterial Biology for the Investigation of the Treatment Effects of Reducing Cholesterol 6-HDL and LDL Treatment Strategies in Atherosclerosis trial; AIM-HIGH, Atherothrombosis Intervention in Metabolic Syndrome With Low HDL/High Triglycerides trial; NIA plaque, The National Institute on Aging Plaque Study; ELIMIT, Effect of Lipid Modification on Peripheral Artery Disease after Endovascular Intervention Trial; HPS2-THRIVE, Heart Protection Study 2–Treatment of HDL to Reduce the Incidence of Vascular Events.
* the Coronary Drug Project (CDP) study included also intermittent cerebral ischemic attacks.

© 2019 D'Andrea E et al. *JAMA Network Open.*

eTable 3. Quality assessment and risk of bias of the studies included in the meta-analyses and meta-regression analysis

Trials (Trial name or First Author)	random sequence generation (selection bias)	allocation concealment (selection bias)	blinding of participants and personnel (performance bias)	blinding of outcome assessors (performance bias)	incomplete outcome data (attrition bias)	selective outcome reporting (reporting bias)	other potential bias	overall assessment: risk of bias
VA drug	?	?	+	?	+	+	+	unclear
CDP	+	+	+	?	+	+	+	low
CLAS I	?	?	+	+	-	-	+	unclear
STOCKHOLM	?	?	-	-	-	?	?	high
FATS	+	+	+	?	+	+	+	low
UCSF-SCOR	+	+	-	-	?	+	+	unclear
HARP	?	?	-	-	+	+	+	high
PAST	?	?	-	-	-	?	+	high
ARBITER - 2	+	+	+	+	-	+	+	low
AFREGS	+	+	+	?	+	+	+	low
Guyton et al	+	+	+	+	-		+	unclear
SANG	?	?	?	?	?	?	-	high
ARBITER-6-HALTS	+	+	-	+	?	-	+	unclear
AIM-HIGH	+	+	+	+	+	+	+	low
NIA plaque	+	+	+	?	-	-	+	unclear
ELIMIT	?	?	+	?	+	+	+	unclear
HPS2-THRIVE	+	+	+	+	+	+	+	low

© 2019 D’Andrea E et al. *JAMA Network Open.*

eFigure 1. Forest plot of meta-analyses on the effect of niacin therapy over time on coronary heart disease mortality, acute coronary syndrome, revascularization procedures, and MACE

Coronary heart disease mortality

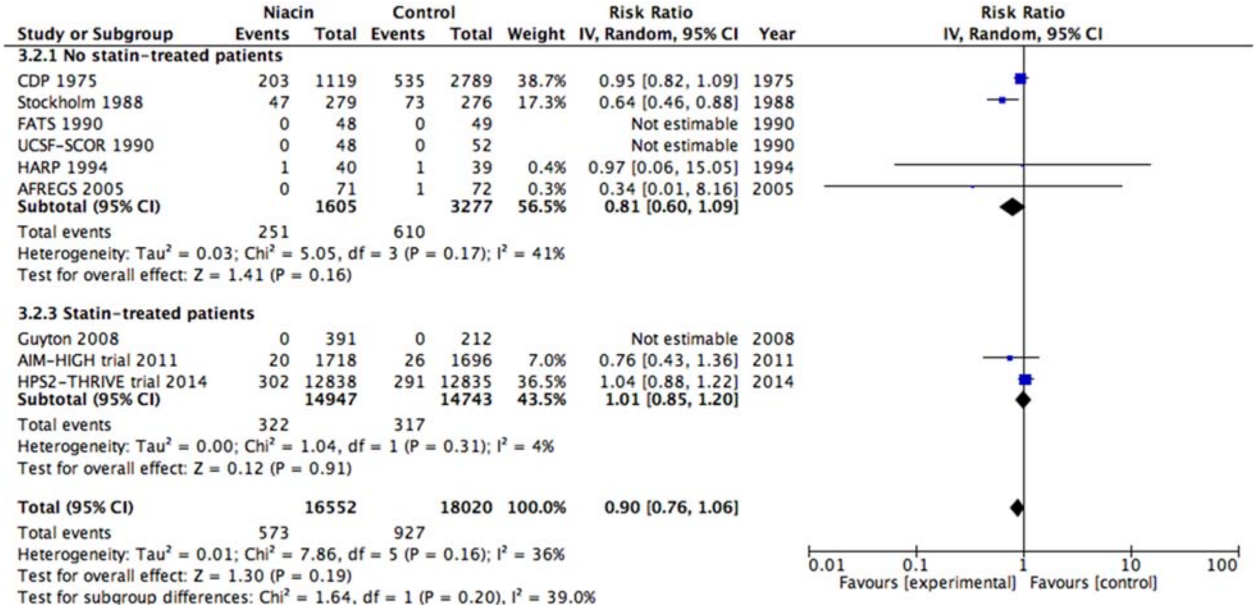

Study or Subgroup	Niacin Events	Niacin Total	Control Events	Control Total	Weight	Risk Ratio IV, Random, 95% CI	Year
**3.2.1 No statin-treated patients**							
CDP 1975	203	1119	535	2789	38.7%	0.95 [0.82, 1.09]	1975
Stockholm 1988	47	279	73	276	17.3%	0.64 [0.46, 0.88]	1988
FATS 1990	0	48	0	49		Not estimable	1990
UCSF-SCOR 1990	0	48	0	52		Not estimable	1990
HARP 1994	1	40	1	39	0.4%	0.97 [0.06, 15.05]	1994
AFREGS 2005	0	71	1	72	0.3%	0.34 [0.01, 8.16]	2005
**Subtotal (95% CI)**		**1605**		**3277**	**56.5%**	**0.81 [0.60, 1.09]**	
Total events	251		610				
Heterogeneity: Tau² = 0.03; Chi² = 5.05, df = 3 (P = 0.17); I² = 41%							
Test for overall effect: Z = 1.41 (P = 0.16)							
**3.2.3 Statin-treated patients**							
Guyton 2008	0	391	0	212		Not estimable	2008
AIM-HIGH trial 2011	20	1718	26	1696	7.0%	0.76 [0.43, 1.36]	2011
HPS2-THRIVE trial 2014	302	12838	291	12835	36.5%	1.04 [0.88, 1.22]	2014
**Subtotal (95% CI)**		**14947**		**14743**	**43.5%**	**1.01 [0.85, 1.20]**	
Total events	322		317				
Heterogeneity: Tau² = 0.00; Chi² = 1.04, df = 1 (P = 0.31); I² = 4%							
Test for overall effect: Z = 0.12 (P = 0.91)							
**Total (95% CI)**		**16552**		**18020**	**100.0%**	**0.90 [0.76, 1.06]**	
Total events	573		927				
Heterogeneity: Tau² = 0.01; Chi² = 7.86, df = 5 (P = 0.16); I² = 36%							
Test for overall effect: Z = 1.30 (P = 0.19)							
Test for subgroup differences: Chi² = 1.64, df = 1 (P = 0.20), I² = 39.0%							

Acute coronary syndrome

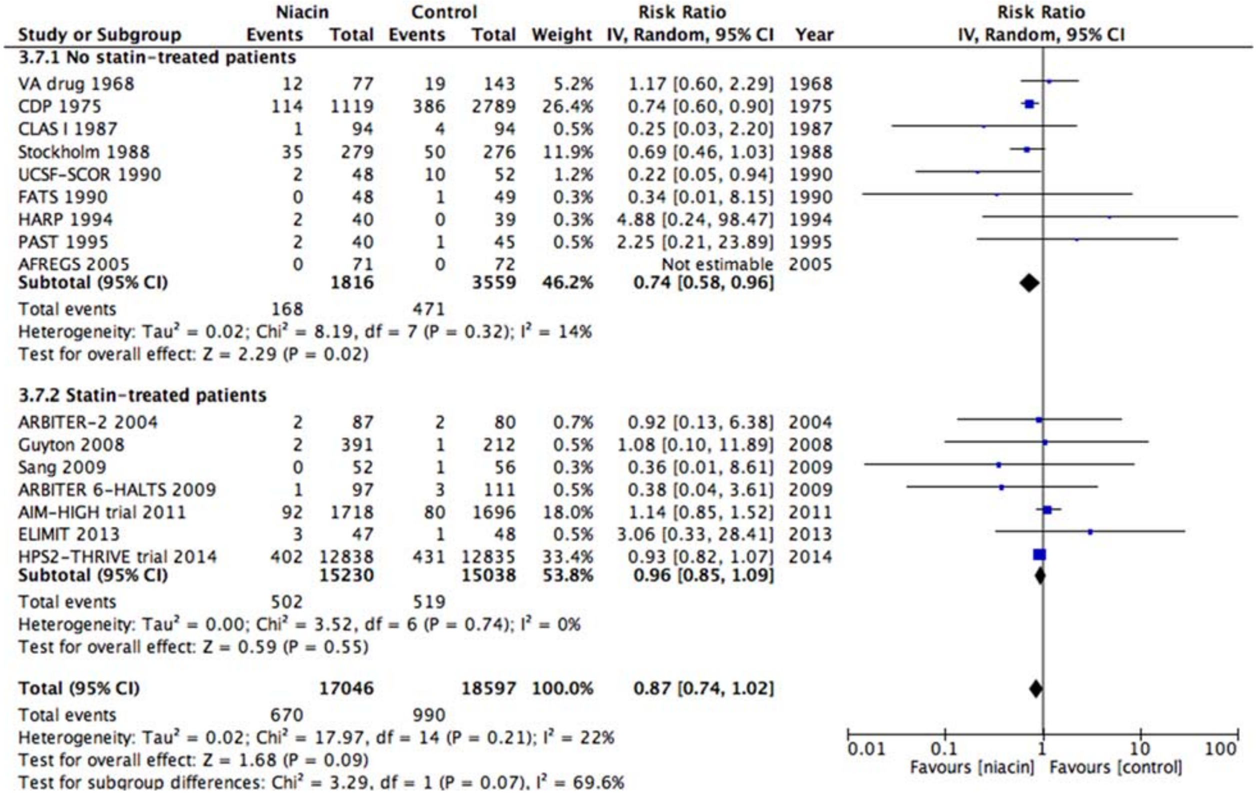

Study or Subgroup	Niacin Events	Niacin Total	Control Events	Control Total	Weight	Risk Ratio IV, Random, 95% CI	Year
**3.7.1 No statin-treated patients**							
VA drug 1968	12	77	19	143	5.2%	1.17 [0.60, 2.29]	1968
CDP 1975	114	1119	386	2789	26.4%	0.74 [0.60, 0.90]	1975
CLAS I 1987	1	94	4	94	0.5%	0.25 [0.03, 2.20]	1987
Stockholm 1988	35	279	50	276	11.9%	0.69 [0.46, 1.03]	1988
UCSF-SCOR 1990	2	48	10	52	1.2%	0.22 [0.05, 0.94]	1990
FATS 1990	0	48	1	49	0.3%	0.34 [0.01, 8.15]	1990
HARP 1994	2	40	0	39	0.3%	4.88 [0.24, 98.47]	1994
PAST 1995	2	40	1	45	0.5%	2.25 [0.21, 23.89]	1995
AFREGS 2005	0	71	0	72		Not estimable	2005
**Subtotal (95% CI)**		**1816**		**3559**	**46.2%**	**0.74 [0.58, 0.96]**	
Total events	168		471				
Heterogeneity: Tau² = 0.02; Chi² = 8.19, df = 7 (P = 0.32); I² = 14%							
Test for overall effect: Z = 2.29 (P = 0.02)							
**3.7.2 Statin-treated patients**							
ARBITER-2 2004	2	87	2	80	0.7%	0.92 [0.13, 6.38]	2004
Guyton 2008	2	391	1	212	0.5%	1.08 [0.10, 11.89]	2008
Sang 2009	0	52	1	56	0.3%	0.36 [0.01, 8.61]	2009
ARBITER 6-HALTS 2009	1	97	3	111	0.5%	0.38 [0.04, 3.61]	2009
AIM-HIGH trial 2011	92	1718	80	1696	18.0%	1.14 [0.85, 1.52]	2011
ELIMIT 2013	3	47	1	48	0.5%	3.06 [0.33, 28.41]	2013
HPS2-THRIVE trial 2014	402	12838	431	12835	33.4%	0.93 [0.82, 1.07]	2014
**Subtotal (95% CI)**		**15230**		**15038**	**53.8%**	**0.96 [0.85, 1.09]**	
Total events	502		519				
Heterogeneity: Tau² = 0.00; Chi² = 3.52, df = 6 (P = 0.74); I² = 0%							
Test for overall effect: Z = 0.59 (P = 0.55)							
**Total (95% CI)**		**17046**		**18597**	**100.0%**	**0.87 [0.74, 1.02]**	
Total events	670		990				
Heterogeneity: Tau² = 0.02; Chi² = 17.97, df = 14 (P = 0.21); I² = 22%							
Test for overall effect: Z = 1.68 (P = 0.09)							
Test for subgroup differences: Chi² = 3.29, df = 1 (P = 0.07), I² = 69.6%							

© 2019 D'Andrea E et al. *JAMA Network Open.*

Fatal and non-fatal stroke

Study or Subgroup	Niacin Events	Niacin Total	Control Events	Control Total	Weight	Odds Ratio M-H, Fixed, 95% CI	Year
**3.4.1 No Statin-treated patients**							
CDP 1975	95	1119	311	2789	24.3%	0.74 [0.58, 0.94]	1975
Stockholm 1988	6	279	5	276	0.7%	1.19 [0.36, 3.95]	1988
FATS 1990	0	48	0	49		Not estimable	1990
UCSF-SCOR 1990	0	48	0	52		Not estimable	1990
AFREGS 2005	0	71	2	72	0.4%	0.20 [0.01, 4.18]	2005
**Subtotal (95% CI)**		**1565**		**3238**	**25.4%**	**0.74 [0.59, 0.94]**	
Total events	101		318				
Heterogeneity: Chi² = 1.32, df = 2 (P = 0.52); I² = 0%							
Test for overall effect: Z = 2.46 (P = 0.01)							
**3.4.2 Statin-treated patients**							
ARBITER-2 2004	0	87	2	80	0.4%	0.18 [0.01, 3.79]	2004
Guyton 2008	0	391	1	212	0.3%	0.18 [0.01, 4.44]	2008
AIM-HIGH trial 2011	27	1718	15	1696	2.2%	1.79 [0.95, 3.38]	2011
NIA plaque 2013	1	59	0	58	0.1%	3.00 [0.12, 75.16]	2013
ELIMIT 2013	2	47	2	48	0.3%	1.02 [0.14, 7.57]	2013
HPS2-THRIVE trial 2014	498	12838	499	12838	71.4%	1.00 [0.88, 1.13]	2014
**Subtotal (95% CI)**		**15140**		**14932**	**74.6%**	**1.02 [0.90, 1.15]**	
Total events	528		519				
Heterogeneity: Chi² = 5.93, df = 5 (P = 0.31); I² = 16%							
Test for overall effect: Z = 0.25 (P = 0.80)							
**Total (95% CI)**		**16705**		**18170**	**100.0%**	**0.95 [0.85, 1.06]**	
Total events	629		837				
Heterogeneity: Chi² = 12.40, df = 8 (P = 0.13); I² = 35%							
Test for overall effect: Z = 0.98 (P = 0.33)							
Test for subgroup differences: Chi² = 5.28, df = 1 (P = 0.02), I² = 81.1%							

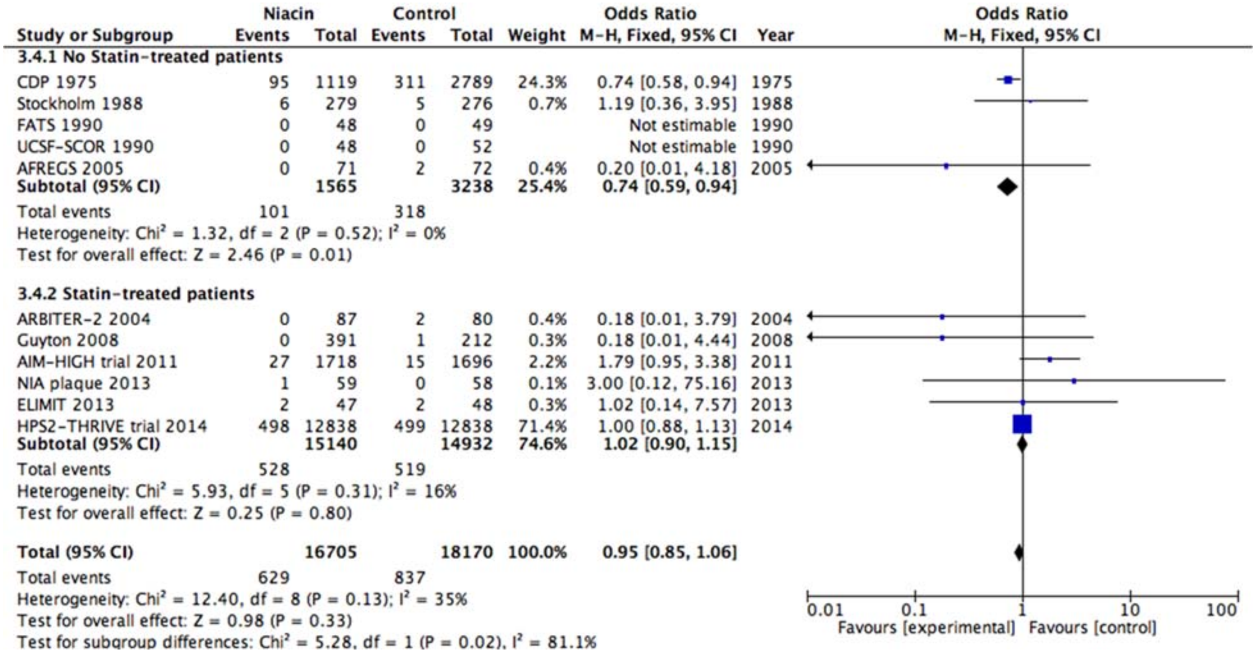

Revascularization procedures

Study or Subgroup	Niacin Events	Niacin Total	Control Events	Control Total	Weight	Risk Ratio IV, Random, 95% CI	Year
**1.7.1 No statin-treated patients**							
CDP 1975	29	1119	137	2789	17.0%	0.53 [0.36, 0.78]	1975
CLAS I 1987	3	94	3	94	1.8%	1.00 [0.21, 4.83]	1987
FATS 1990	2	48	7	52	1.9%	0.31 [0.07, 1.42]	1990
HARP 1994	2	40	3	39	1.5%	0.65 [0.11, 3.68]	1994
PAST 1995	3	40	6	45	2.5%	0.56 [0.15, 2.10]	1995
AFREGS 2005	4	71	12	72	3.6%	0.34 [0.11, 1.00]	2005
**Subtotal (95% CI)**		**1412**		**3091**	**28.2%**	**0.51 [0.37, 0.72]**	
Total events	43		168				
Heterogeneity: Tau² = 0.00; Chi² = 1.79, df = 5 (P = 0.88); I² = 0%							
Test for overall effect: Z = 3.92 (P < 0.0001)							
**1.7.2 Statin-treated patients**							
ARBITER-2 2004	1	87	4	80	1.0%	0.23 [0.03, 2.01]	2004
ARBITER 6-HALTS 2009	0	97	3	111	0.5%	0.16 [0.01, 3.12]	2009
Sang 2009	2	52	1	56	0.8%	2.15 [0.20, 23.05]	2009
AIM-HIGH trial 2011	167	1718	168	1696	29.5%	0.98 [0.80, 1.20]	2011
NIA plaque 2013	5	59	2	58	1.7%	2.46 [0.50, 12.16]	2013
ELIMIT 2013	2	47	1	48	0.8%	2.04 [0.19, 21.77]	2013
HPS2-THRIVE trial 2014	807	12838	897	12835	37.4%	0.90 [0.82, 0.99]	2014
**Subtotal (95% CI)**		**14898**		**14884**	**71.8%**	**0.91 [0.84, 0.99]**	
Total events	984		1076				
Heterogeneity: Tau² = 0.00; Chi² = 5.86, df = 6 (P = 0.44); I² = 0%							
Test for overall effect: Z = 2.11 (P = 0.03)							
**Total (95% CI)**		**16310**		**17975**	**100.0%**	**0.79 [0.64, 0.98]**	
Total events	1027		1244				
Heterogeneity: Tau² = 0.03; Chi² = 18.50, df = 12 (P = 0.10); I² = 35%							
Test for overall effect: Z = 2.12 (P = 0.03)							
Test for subgroup differences: Chi² = 10.85, df = 1 (P = 0.0010), I² = 90.8%							

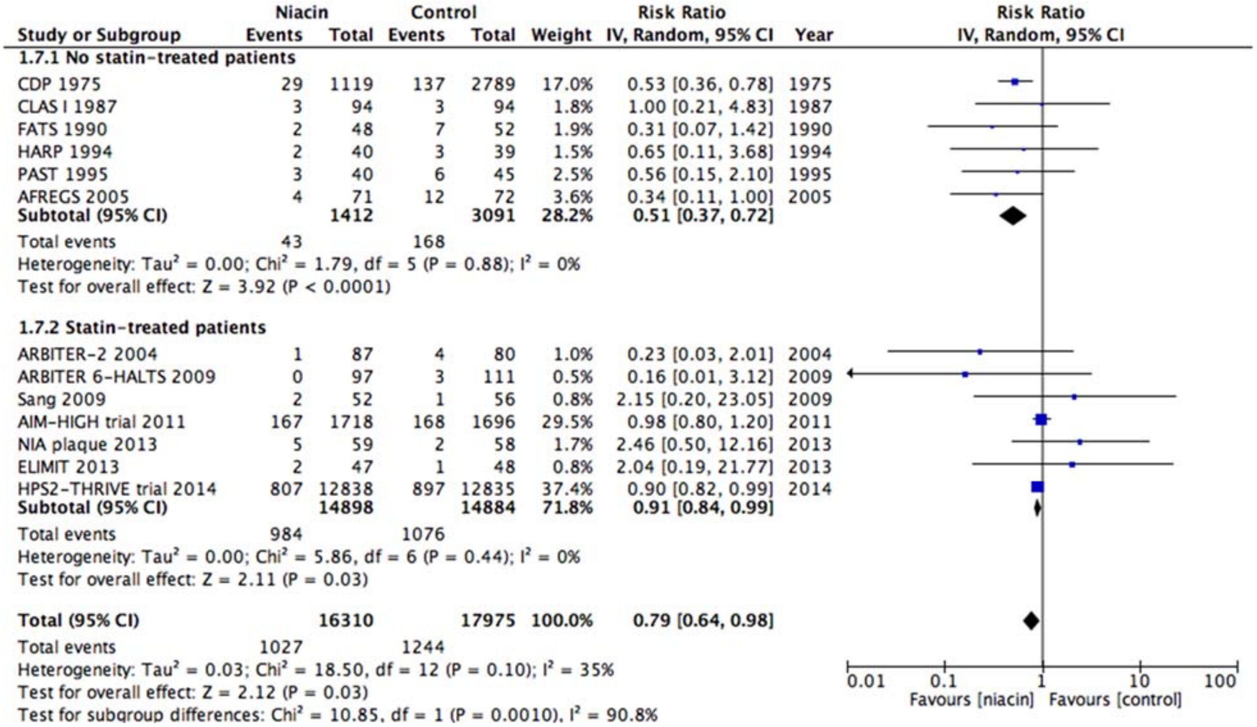

© 2019 D'Andrea E et al. *JAMA Network Open.*

Major adverse cardiac events (MACE)

Study or Subgroup	Niacin Events	Niacin Total	Control Events	Control Total	Weight	Risk Ratio IV, Random, 95% CI	Year
**1.8.1 No statin-treated patients**							
VA drug 1968	33	77	60	143	11.3%	1.02 [0.74, 1.41]	1968
CDP 1975	914	1119	2333	2789	26.1%	0.98 [0.95, 1.01]	1975
CLAS I 1987	25	94	25	94	6.7%	1.00 [0.62, 1.61]	1987
Stockholm 1988	90	279	129	276	16.7%	0.69 [0.56, 0.85]	1988
UCSF-SCOR 1990	0	48	1	49	0.2%	0.34 [0.01, 8.15]	1990
FATS 1990	2	48	11	52	0.9%	0.20 [0.05, 0.84]	1990
HARP 1994	6	40	10	39	2.2%	0.58 [0.24, 1.45]	1994
PAST 1995	7	40	10	45	2.5%	0.79 [0.33, 1.87]	1995
AFREGS 2005	9	71	19	72	3.4%	0.48 [0.23, 0.99]	2005
**Subtotal (95% CI)**		**1816**		**3559**	**70.0%**	**0.81 [0.66, 1.00]**	
Total events	1086		2598				
Heterogeneity: Tau² = 0.04; Chi² = 20.24, df = 8 (P = 0.009); I² = 60%							
Test for overall effect: Z = 1.94 (P = 0.05)							
**1.8.2 Statin-treated patients**							
ARBITER-2 2004	3	87	7	80	1.1%	0.39 [0.11, 1.47]	2004
Guyton 2008	4	391	4	212	1.0%	0.54 [0.14, 2.15]	2008
ARBITER 6-HALTS 2009	2	97	9	111	0.9%	0.25 [0.06, 1.15]	2009
Sang 2009	3	52	4	56	0.9%	0.81 [0.19, 3.44]	2009
AIM-HIGH trial 2011	282	1718	274	1696	20.3%	1.02 [0.87, 1.18]	2011
ELIMIT 2013	10	97	9	111	2.5%	1.27 [0.54, 3.00]	2013
NIA plaque 2013	6	59	3	58	1.1%	1.97 [0.52, 7.49]	2013
HPS2-THRIVE trial 2014	10	47	6	48	2.2%	1.70 [0.67, 4.31]	2014
**Subtotal (95% CI)**		**2548**		**2372**	**30.0%**	**0.98 [0.71, 1.34]**	
Total events	320		316				
Heterogeneity: Tau² = 0.04; Chi² = 8.50, df = 7 (P = 0.29); I² = 18%							
Test for overall effect: Z = 0.14 (P = 0.89)							
**Total (95% CI)**		**4364**		**5931**	**100.0%**	**0.88 [0.76, 1.01]**	
Total events	1406		2914				
Heterogeneity: Tau² = 0.02; Chi² = 29.07, df = 16 (P = 0.02); I² = 45%							
Test for overall effect: Z = 1.76 (P = 0.08)							
Test for subgroup differences: Chi² = 0.94, df = 1 (P = 0.33), I² = 0%							

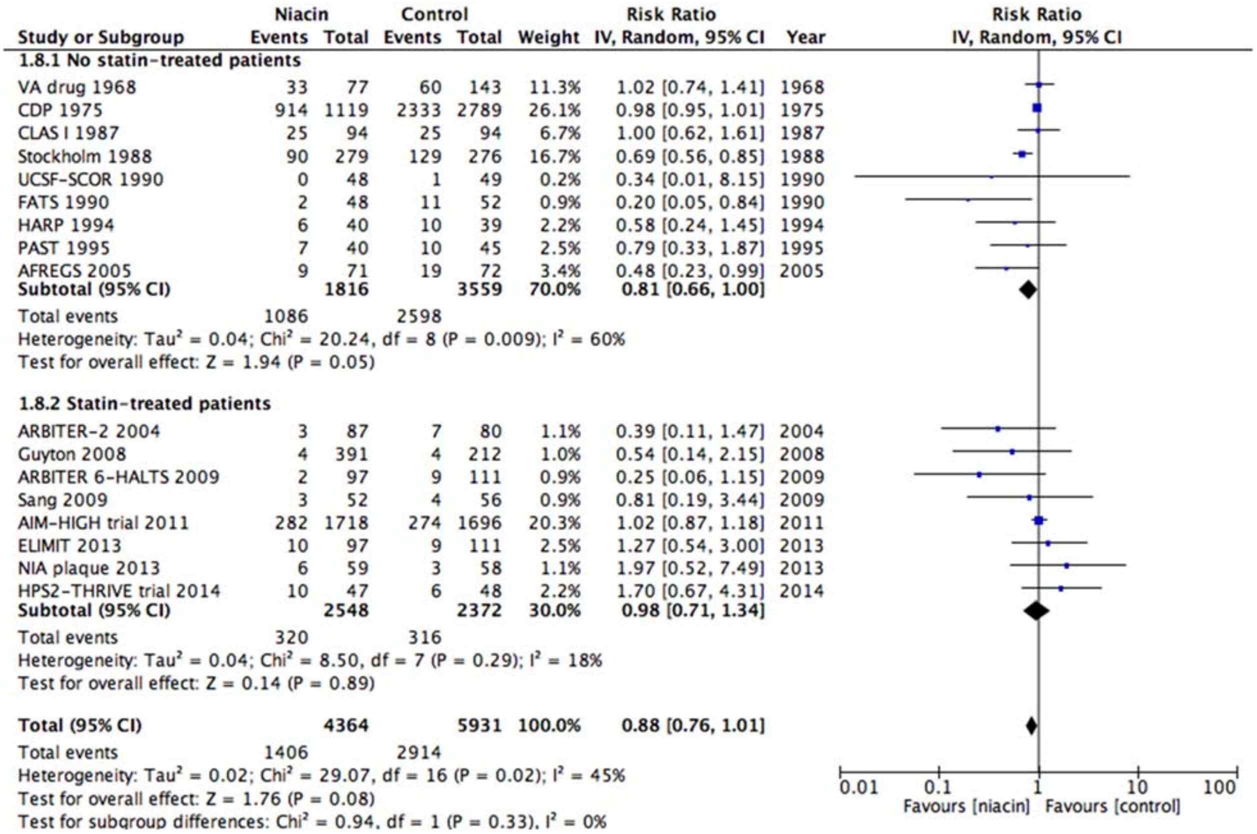

MACE, major adverse cardiovascular events; VA drug, Veterans Affairs drug study; CDP, Coronary Drug Project; CLAS I, Cholesterol-Lowering Atherosclerosis Study I; STOCKHOLM, Stockholm Ischaemic Heart Disease Secondary prevention study; FATS, Familial Atherosclerosis Treatment Study; UCSF-SCOR, University of California San Francisco - Arteriosclerosis Specialized Center of Research Intervention Trial; HARP, Heart and Renal Protection study; PAST, Prevenzione Aterosclerosi Studio Torino; ARBITER-2, Arterial Biology for the Investigation of the Treatment Effects of Reducing Cholesterol trial; AFREGS, Armed Forces Regression Study; ARBITER 6-HALTS, Arterial Biology for the Investigation of the Treatment Effects of Reducing Cholesterol 6-HDL and LDL Treatment Strategies in Atherosclerosis trial; AIM-HIGH, Atherothrombosis Intervention in Metabolic Syndrome With Low HDL/High Triglycerides trial; NIA plaque, The National Institute on Aging Plaque Study; ELIMIT, Effect of Lipid Modification on Peripheral Artery Disease after Endovascular Intervention Trial; HPS2-THRIVE, Heart Protection Study 2–Treatment of HDL to Reduce the Incidence of Vascular Events.

© 2019 D'Andrea E et al. *JAMA Network Open.*

eFigure 2. Forest plot of cumulative meta-analyses on the effect of niacin therapy over time on the CVD mortality, coronary heart disease mortality, acute coronary syndrome, revascularization procedures, and MACE

CVD mortality

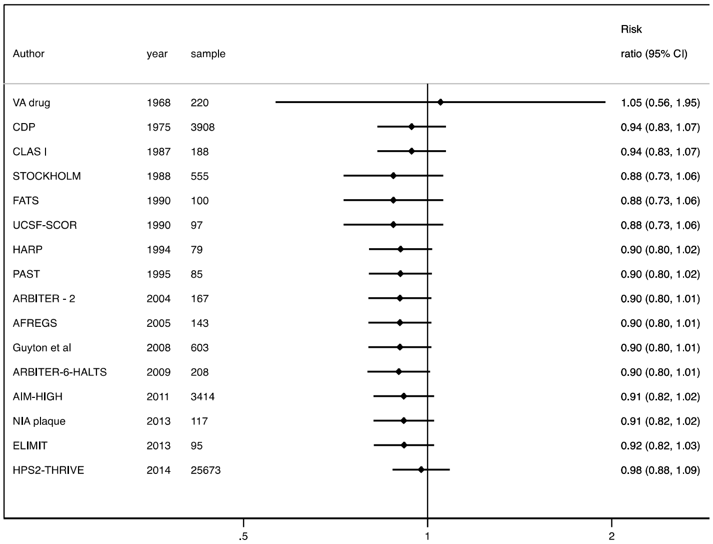

Coronary heart disease mortality

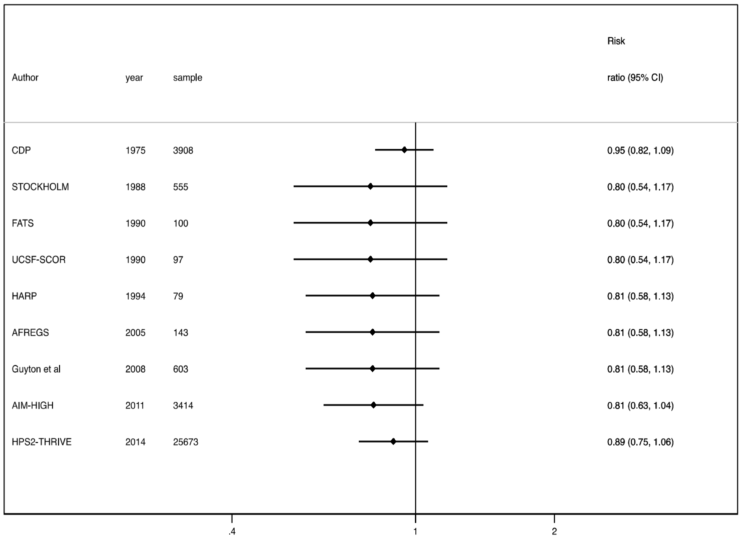

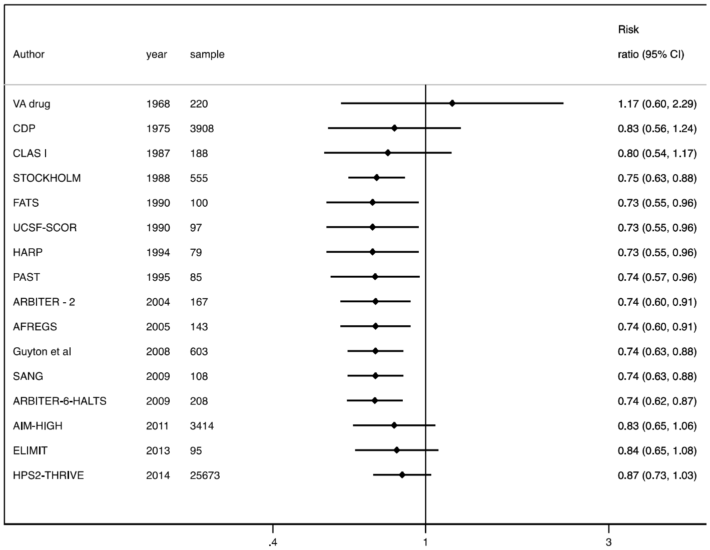

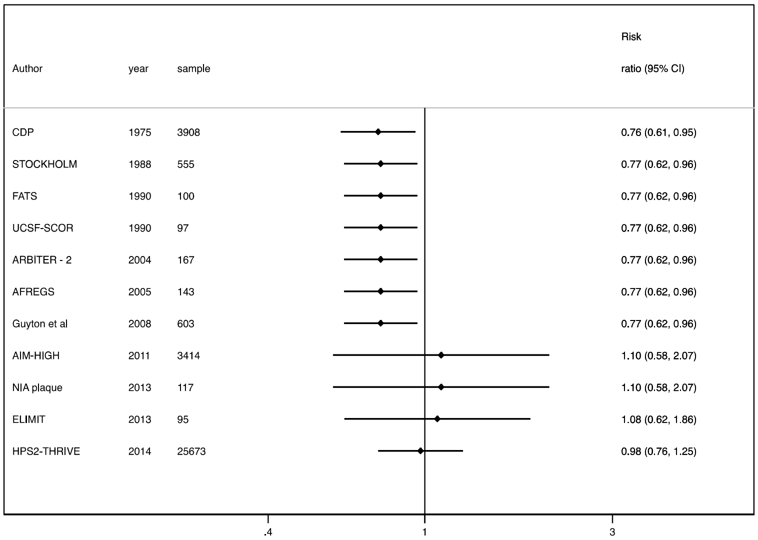

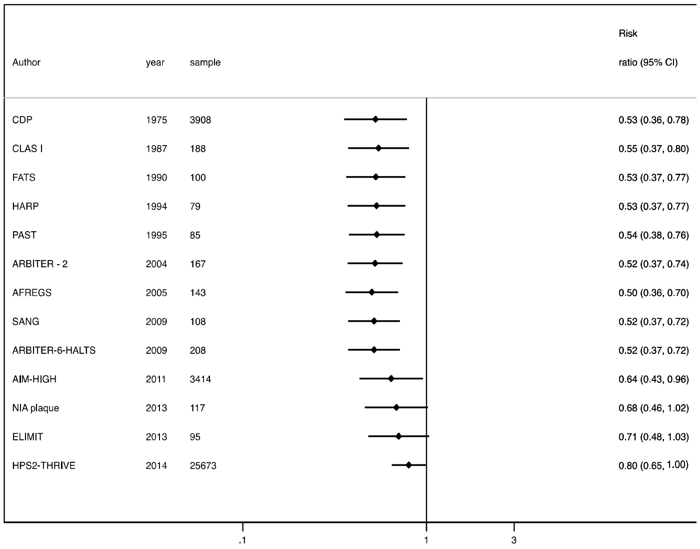

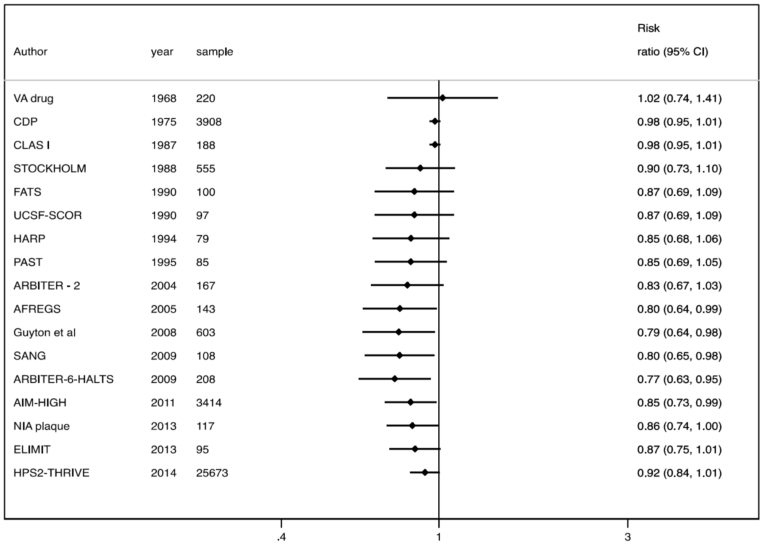

CVD, cardiovascular disease; MACE, major adverse cardiovascular events; VA drug, Veterans Affairs drug study; CDP, Coronary Drug Project; CLAS I, Cholesterol-Lowering Atherosclerosis Study I; STOCKHOLM, Stockholm Ischaemic Heart Disease Secondary prevention study; FATS, Familial Atherosclerosis Treatment Study; UCSF-SCOR, University of California San Francisco - Arteriosclerosis Specialized Center of Research Intervention Trial; HARP, Heart and Renal Protection study; PAST, Prevenzione Aterosclerosi Studio Torino; ARBITER-2, Arterial Biology for the Investigation of the Treatment Effects of Reducing Cholesterol trial; AFREGS, Armed Forces Regression Study; ARBITER 6-HALTS, Arterial Biology for the Investigation of the Treatment Effects of Reducing Cholesterol 6-HDL and LDL Treatment Strategies in Atherosclerosis trial; AIM-HIGH, Atherothrombosis Intervention in Metabolic Syndrome With Low HDL/High Triglycerides trial; NIA plaque, The National Institute on Aging Plaque Study; ELIMIT, Effect of Lipid Modification on Peripheral Artery Disease after Endovascular Intervention Trial; HPS2-THRIVE, Heart Protection Study 2–Treatment of HDL to Reduce the Incidence of Vascular Events.

© 2019 D’Andrea E et al. *JAMA Network Open.*
